# A Novel *In Silico* Benchmarked Pipeline Capable of Complete Protein Analysis: A Possible Tool for Potential Drug Discovery

**DOI:** 10.3390/biology10111113

**Published:** 2021-10-28

**Authors:** D. D. B. D. Perera, K. Minoli L. Perera, Dinithi C. Peiris

**Affiliations:** 1 Department of Zoology, Faculty of Applied Sciences, University of Sri Jayewardenepura, Nugegoda 10250, Sri Lanka; mperera95826@gmail.com; 2 Genetics & Molecular Biology Unit (Center for Biotechnology), Department of Zoology, Faculty of Applied Sciences, University of Sri Jayewardenepura, Nugegoda 10250, Sri Lanka

**Keywords:** virtual screening, therapeutic targets, protein modulation, trifecta analysis

## Abstract

**Simple Summary:**

Protein interactions govern the majority of an organism’s biological processes. Therefore, to fully understand the functionality of an organism, we must know how proteins work at a molecular level. This study assembled a protocol that enables scientists to construct a protein’s tertiary structure easily and subsequently to investigate its mechanism and function. Each step involved in prediction, validation, and functional analysis of a protein is crucial to obtain an accurate result. We have dubbed this the trifecta analysis. It was clear early in our research that no single study in the literature had previously encompassed the complete trifecta analysis. In particular, studies that recommend free, open-source tools that have been benchmarked for each step are lacking. The present study ensures that predictions are accurate and validated and will greatly benefit new and experienced scientists alike in obtaining a strong understanding of the trifecta analysis, resulting in a domino effect that could lead to drug development.

**Abstract:**

Current *in silico* proteomics require the trifecta analysis, namely, prediction, validation, and functional assessment of a modeled protein. The main drawback of this endeavor is the lack of a single protocol that utilizes a proper set of benchmarked open-source tools to predict a protein’s structure and function accurately. The present study rectifies this drawback through the design and development of such a protocol. The protocol begins with the characterization of a novel coding sequence to identify the expressed protein. It then recognizes and isolates evolutionarily conserved sequence motifs through phylogenetics. The next step is to predict the protein’s secondary structure, followed by the prediction, refinement, and validation of its three-dimensional tertiary structure. These steps enable the functional analysis of the macromolecule through protein docking, which facilitates the identification of the protein’s active site. Each of these steps is crucial for the complete characterization of the protein under study. We have dubbed this process the trifecta analysis. In this study, we have proven the effectiveness of our protocol using the cystatin C and AChE proteins. Beginning with just their sequences, we have characterized both proteins’ structures and functions, including identifying the cystatin C protein’s seven-residue active site and the AChE protein’s active-site gorge via protein–protein and protein–ligand docking, respectively. This process will greatly benefit new and experienced scientists alike in obtaining a strong understanding of the trifecta analysis, resulting in a domino effect that could expand drug development.

## 1. Introduction

In the scientific endeavor of understanding the mechanisms of an organism’s biology, the study of proteins, which are key elements in several cellular activities, is vital. Proteins play both structural and functional roles within the organization of a cell [1]. A protein’s characteristic and functionally related 3D structure originates from a simple but unique amino acid sequence [2]. Understanding a novel amino acid sequence that codes for a particular protein begins with identifying the sequence’s relatedness to other proteins with a similar polypeptide chain [2]. Sequencing is followed by the characterization and functional analysis of the protein by studying its three-dimensional tertiary or even quaternary structure. For this trifecta analysis of identification, characterization, and function prediction, many bioinformatics tools exist [3]. However, a single protocol that encompasses all three processes utilizing the best-suited tools based on efficiency and accuracy is lacking [4]. The present study aims to fill this gap by using a series of open-source *in silico* tools to achieve this goal economically, thus creating a tested and proven pathway for sound protein analysis.

With the completion of the human genome project and the genomes of entire organisms being decoded almost every day, the gap between proteins with known sequences and those with experimentally validated structures and functions has increased rapidly [5]. Therefore, the requirement for a protocol capable of converting genomic data into functional information has become more crucial [3]. Several experimental procedures exist today for the determination of a protein’s tertiary structure. X-ray crystallography, cryo-EM, and nuclear magnetic resonance are some of the most commonly used pathways. However, the main shortcomings of these methods are that they lack efficiency and are highly costly [1]. These drawbacks prevent a significant portion of the scientific community from conducting comprehensive studies of a protein’s structure and function, primarily due to lack of access to such specialized and costly equipment.

The process of protein identification is the first step in any protein study. Following protein identification, recognition of its relatedness to other closely related proteins via conserved sequence motifs and sequence similarity is crucial [6]. This step of the analysis helps reveal conserved functional domains. These conserved regions across species have been found through experimentation to contribute to the macromolecule’s function [7]. *In silico* tools for phylogenetic analysis and multiple sequence alignment (MSA) cater for this requirement.

With the advancements in DNA sequencing and the increase in the number of predicted amino acid sequences, there is a serious need for the comprehensive analysis of these novel proteins in the study of proteomics. As proven through experimentation, a complete analysis requires identification of the amino acid sequence within a non-redundant database, then characterization and functional prediction through protein modeling techniques [8]. However, in the current environment of bioinformatics, a single, open-source, and complete pipeline that caters for all of these functions is absent. 

The present study attempts to fill this gap utilizing three examples. The first example involves the amino acid sequence of *Danio rerio,* which codes for the protease-inhibiting cystatin C protein and its interactions with human cathepsin proteases. The second and third examples involve the AChE protein-coding sequences of *Homo sapiens* and *Rattus norvegicus* and their interactions with the organophosphate echothiophate.

The zebrafish, otherwise known as *Danio rerio,* is a model organism of vertebrate development and is of considerable scientific interest, especially in modeling human biology and disease [9]. Cystatin C is a type 2 cystatin protein present in all vertebrate organisms. It is known for being a competitive protease inhibitor of papain-like proteases such as cathepsin B, H, L, and S, competitively blocking the cysteine-active site of the cathepsin proteins [10]. Due to its function as a competitive inhibitor of proteases, it plays a crucial role in regulating certain diseases caused by cathepsin overexpression, such as atherosclerosis and metastasis of cancer cells [11]. 

Organophosphates such as echothiophate could inhibit AChE activity. The mechanism of AChE inhibition is well known, making this an ideal candidate for benchmarking the proposed pathway. This study focused on the AChE proteins of *Homo sapiens* (hAChE) and *Rattus norvegicus* (rAChE). AChE proteins have been predicted to possess an active-site gorge with a tryptophan residue, and echothiophate is known to bind at this active site [12]. The inhibitory mechanisms are essential for identifying the evolutionarily conserved active sites [13] successfully. 

The hAChE macromolecule has already been modeled and is readily available in the PDB data bank [14]. The primary reason for selecting hAChE was to quantitatively identify the accuracy of the proposed pipeline for predicting the actual structure. In contrast, the rAChE molecule provided us with an opportunity to predict another novel protein and comparatively analyze it against its human counterpart.

The primary focus in selecting the cystatin C protein encoded by zebrafish and the AChE proteins was to benchmark the timeline with a multitude of queries and provide a wealth of information to the scientific community. The selection of an immune-related protein provided an opportunity to benchmark all the features of the proposed pipeline. Here we report novel *in silico* tools to generate valid protein models, prove their functionality through molecular docking techniques, and discover the immune potential against diseases in the human body by using gene–gene interaction mapping. The study aims to develop an economical *in silico* approach to analyzing the structure and functionality of novel proteins with a comprehensive revelation of their clinical significance.

## 2. Materials and Methods

**Figure 1:**
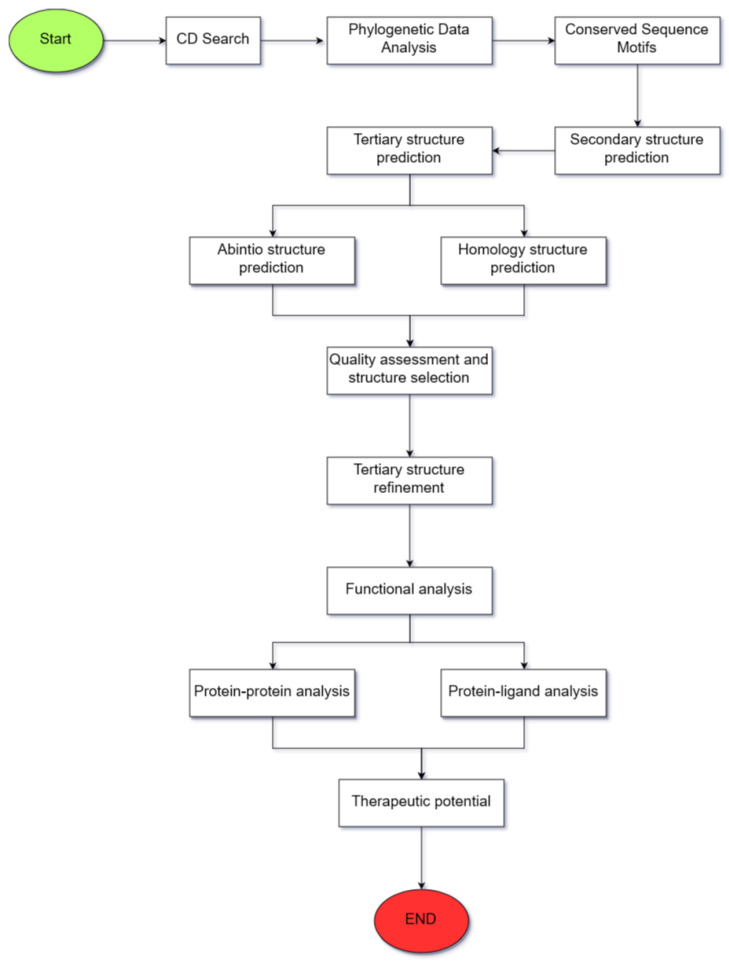
A graphical representation of the proposed methodology summarizing the sequence of steps that should be followed.

The proposed methodology (Figure 1) aims to provide the reader with a guided protocol for protein analysis. Its foundation is the amino acid sequence from which the translated protein’s structure and function are analyzed. We emphasize that for a complete protein study, the three processes of (1) prediction of the translated protein and its characterization, (2) prediction of the protein’s tertiary structure and validation of its structure, and (3) functional analysis of the protein must be conducted. 

In the present study, we have detailed the steps required for such an analysis. We have validated our pipeline through a series of analyses using a selection of computational tools. It is possible to replace these tools with others as required by the user. Our selection of these tools is based on their time-tested reliability, the fact that they are free to use, and the fact that they can be used on general hardware.

We proved the robustness of our proposed pipeline using a series of use cases. In this study, we detailed the use of the complete pipeline for three proteins, namely, cystatin C from *D. rerio* and acetylcholinesterase from humans (hAChE) and *Rattus norvegicus* (rAChE). The use of these three proteins enabled the testing of the protocol in multiple different situations that may arise in the analysis of the structure and function of a protein. The cystatin C of *D. rerio* and the rAChE proteins enabled the protocol to conduct the trifecta analysis of novel structures and test their functions in protein–protein and protein–ligand interactions. The hAChE example enabled the methodology to be comparatively tested against a protein of known structure. The actual structure of hAChE is readily available in the PDB data bank (PDB ID: 4PQE). The analyses of both cystatin C and AChE are novel. These findings are unique to this work, and we believe they help to explain the power of a standardized protocol. However, these predictions still need to be validated via experimentation. 

To further prove the validity of our work, we conducted protein–ligand and protein–protein replication studies. To analyze protein–ligand interactions, we analyzed the survivin protein and 4-hydroxy pyridine 1-oxide pyridin-4-ol 1-oxide ligand, and the more well-known hemoglobin protein and haem ligand [15,16]. These replication studies have been proven through experimentation, and we believe that our results confirm the reliability and ad hoc nature of the proposed process. 

### 2.1. Validation of the Selection of the Proposed Computational Tools

Two primary concerns were reliability and accessibility. We wanted to ensure the selected tools were time-tested and freely available to all users. These tools guide the use of our trifecta analysis but can be changed as the user deems fit. 

For functions involving protein characterization, such as identifying conserved domains and collecting sequences for analysis, we chose NCBI tools such as their CD-Search and database [17]. The NCBI model was developed specifically for genomic sequences. Its services and tools include stringent quality assurance protocols. These powerful tools and the database’s long-lasting reliability have secured its place as the industry standard in genomics [18,19,20,21,22].

MEGA software was selected to fulfill our phylogenetic needs. MEGA is equipped with a user-friendly graphical user interface (GUI) that simplifies complex phylogenetic analyses. It has sound statistical models that ensure the accuracy of its analyses. Additionally, it can be run on general-purpose computer hardware, in contrast to its counterparts such as PAML and BEAST, which may require high-performance computing (HPC). PAML and BEAST are known to require a steep learning curve. PAML, with its command-line-based usage, and BEAST require a sound knowledge of Bayesian statistics and MCMC methodologies [23,24,25], both of which can be overwhelming for new users.

To predict the proteins’ tertiary structures, we selected Zhang Lab’s I-TASSER and QUARK servers. Both services are available to academics and have quick turnaround times (usually three days). I-TASSER was the number one server used in all CASP experiments from CASP7 to CASP14, and QUARK was ranked number one in both CASP9 and CASP10 experiments [26,27,28]. Recently they have been overtaken by Google’s DeepMind AlphaFold 2. However, it should be noted that AlphaFold 2 is unavailable at this time for public use [29].

The selected docking software was Hex 8.0.0 for protein–protein docking and AutoDock Vina for protein–ligand docking [30,31,32]. Hex 8.0.0 is readily available software specialized for protein–protein docking, validated by the CAPRI blind docking experiments. Hex 8.0.0 is widely used software that runs independently on general-purpose computers without the use of expensive servers [33,34]. Alternatives to Hex, such as HDOCK and PatchDock 1.0 rely on the submission of experiments to servers, and so have relatively long turnaround times of a few days [34]. Hex 8.0.0 is capable of running most analyses in a few minutes. AutoDock Vina is the most widely used protein–ligand docking software to date. Its free and open-source license, coupled with its speed and user-friendliness, has made it the tool of choice for novices and experts alike. AutoDock Vina has proven its robustness against its counterparts such as GOLD and its predecessor AutoDock4, and in certain scenarios has surpassed commercial software such as Glide in its prediction capabilities [32,35,36]. 

### 2.2. Identification of Conserved Domains

The amino acid sequence coding for the putative cystatin C protein of *D. rerio* (Accession No. AAZ29462.1), rat AChE (AAH94521.1), and human AChE (AAA68151.1) were identified using the NCBI database. The conserved functional domains present in the translated amino acid sequences were identified using the conserved domain search service (CD-Search) in NCBI. The predicted protein was annotated, and a graphical summary was obtained [19].

### 2.3. Phylogenetic Data Analysis

The protein families evolutionarily related to the predicted protein of *D. rerio* were identified using the HUGO Gene Nomenclature Committee search tool. The related proteins were searched for in the NCBI database, and the amino acid sequence was extracted in the FASTA format. It was ensured that at least one amino acid sequence was obtained for each of the five vertebrate classes: Pisces, Amphibia, Reptilia, Aves, and Mammalia. These amino acid sequences were subsequently aligned using ClustalW with the MEGA6 software, and a phylogenetic tree was obtained using the neighbor-joining statistical method.

### 2.4. Identification of Conserved Sequence Motifs

The translated protein sequences were subjected to multiple sequence alignment by ClustalW, performed using the program Unipro UGENE [35]. The analyses were conducted using ten of the most closely related amino acid sequences to identify sequence motifs that have been evolutionarily conserved. 

### 2.5. Amino Acid Sequence and Secondary Structure Characterization

The secondary structure expressed by the amino acid sequence was predicted using the JPred secondary structure prediction server [36]. 

### 2.6. Tertiary Structure Prediction

The 3D structure of the protein was predicted using two main systems. Homology prediction of the protein was conducted using the I-TASSER online software [5]. *Ab initio* model prediction was performed using the QUARK server [27]. However, *ab initio* modeling was not utilized to predict the AChE molecules due to the limitation of the server to 200 amino acids. The predicted 3D structures were visualized using PyMOL visualization software [37]. Cystatin C proteins are required to possess a series of evolutionarily conserved structural features. The characteristic features include a short and a long alpha-helix lying across a five-stranded antiparallel beta-sheet with two disulfide bridges. The predicted structure possessing all of these features was selected as the most accurate model. The tertiary structure of AChE consisted of a centrally placed mixture of beta-sheets surrounded by 15 alpha-helices [13]. 

### 2.7. Quality Assessment of the Predicted Structure 

The successfully predicted structure was evaluated for its overall quality and stability, to identify any errors that may be present. The general model quality and the local model quality were assessed using the ProSA-web service [38]. The stability and stereochemistry of the structure were evaluated by generating a Ramachandran plot using the PROCHECK software [39].

### 2.8. Tertiary Structure Refinement 

The overall tertiary model quality was improved using the ReFOLD online software. The software was utilized to assess the global model quality, the overall structural improvement, and the accuracy [40]. The refined structure was then subjected to validation and quality assessment to ensure it met all the protein requirements.

### 2.9. Surface Analysis of the Tertiary Structure

The surface of the protein structure was analyzed for potential active binding sites by examining the distribution of electrostatic charges and hydrophobic amino acids using the protein surface analyzer tool from the Maestro BioLuminate 2.8 software [41]. 

### 2.10. Functional Analysis through Molecular Docking

In the functional analysis of a protein, it is essential to determine the type of interactions occurring in the target macromolecule. The following protocol addresses the most common protein interactions, namely protein–ligand [15] and protein–protein interactions [42]. For protein–protein interactions, the cystatin C protein, along with its numerous cathepsin substrates, was considered. Rigid-body protein–protein docking was conducted to analyze the functional effectiveness of the cystatin C protein produced by *D. rerio* as a cysteine protease inhibitor. Hex 8.0.0 CUDA was used, together with fast Fourier transform (FFT) correlation techniques [43]. The software was configured to search for 2000 solutions, and the top 100 were extracted. The required protein models were obtained from the Protein Data Bank (RCSB). After the docking process, the interacting amino acids of the proteins in each protein–protein interaction were analyzed using Maestro BioLuminate 2.8 software and LigPlot+ [44,45].

For successful inhibition of the cysteine protease, the cystatin C protein had to block the cysteine active site of the target protein with an excellent negative binding energy. The protein was first subjected to molecular docking with papain (PDB ID: 9PAP), which is known to be inhibited by all cystatins. The docking was followed by papain-like proteases, namely, cathepsin B (PDB ID: 2IPP), cathepsin H (PDB ID: 8PCH), cathepsin L1 (PDB ID: 2Y2J, and cathepsin S (PDB ID: 2FRQ). The proteases have been proven to be actively inhibited by cystatin C [46]. 

AutoDock Vina was chosen for the protein–ligand docking software. The protein–ligand substrate consisted of AChE and the organophosphate echothiophate (PubChem CID: 10548). A negative control, imidazole (PubChem CID: 795), was used to reduce the risk of false positives and ensure proper testing. To further prove the reliability of our selected software, AutoDock Vina, two replication analyses were conducted. The first, between the survivin protein (PDB ID: 1XOX) and 4-hydroxy pyridine 1-oxide pyridin-4-ol 1-oxide ligand (PubChem ID: 23321) was initially conducted by Heendeniya et al. [15], and that between the hemoglobin protein (PDB ID: 1GZX) and haem ligand (PDB Chemical ID: H.E.M.) was initially conducted by Paoli et al. [16].

In AutoDock, the protein was prepared by removing water molecules followed by the addition of hydrogens and merging of non-polar hydrogens. Finally, computation of the charges was conducted using the Gasteiger function. Grid preparation was performed for the potential active sites. In AutoDock, the ligand was prepared by assigning a torsion tree and detecting the root. AutoDock Vina provided the nine best poses with default exhaustiveness. Subsequently, a 2D analysis of the protein–ligand complexes was performed using Discovery Studio 2017 or LigPlot+ [44].

### 2.11. Analysis of the Therapeutic Potential 

Clinically significant pathways involving cathepsin B, L1, and S overexpression in the human body were identified through gene–gene interaction mapping. However, cathepsin H was excluded due to the absence of its 3D tertiary structure of human origin. The GeneMANIA database coupled with the Cystoscape software was used to identify these protein interactions. The interactions were separated into physical interactions, co-expression, and co-localization [47]. The selected natural substrates that were subjected to degradation by cathepsin overexpression resulting in a clinical response were subjected to molecular docking with the respective cathepsin. The binding energies were compared to that of cystatin C produced by *D. rerio*. If the cystatin C of *D. rerio* had a more feasible binding energy (a lower energy value implies a stronger binding affinity), it was estimated to have clinical potential as a drug to solve the cathepsin overexpression.

## 3. Results

### 3.1. Identification of Conserved Domains

The initial analysis of the cystatin C amino acid sequence obtained from the NCBI database (Accession No. AAZ29462.1) contained a cystatin protein-coding domain complete with an N-terminal glycine and a QxVxG sequence motif. 

The analysis of the AChE of rAChE (AAH94521.1) and hAChE (AAA68151.1) amino acid sequences showed an AB-hydrolase superfamily, complete with a substrate-binding pocket and a catalytic triad. AChE is a member of the AB-hydrolase superfamily, referred to as the alpha/beta hydrolase fold family of enzymes [48].

### 3.2. Phylogenetic Data Analysis

**Figure 2:**
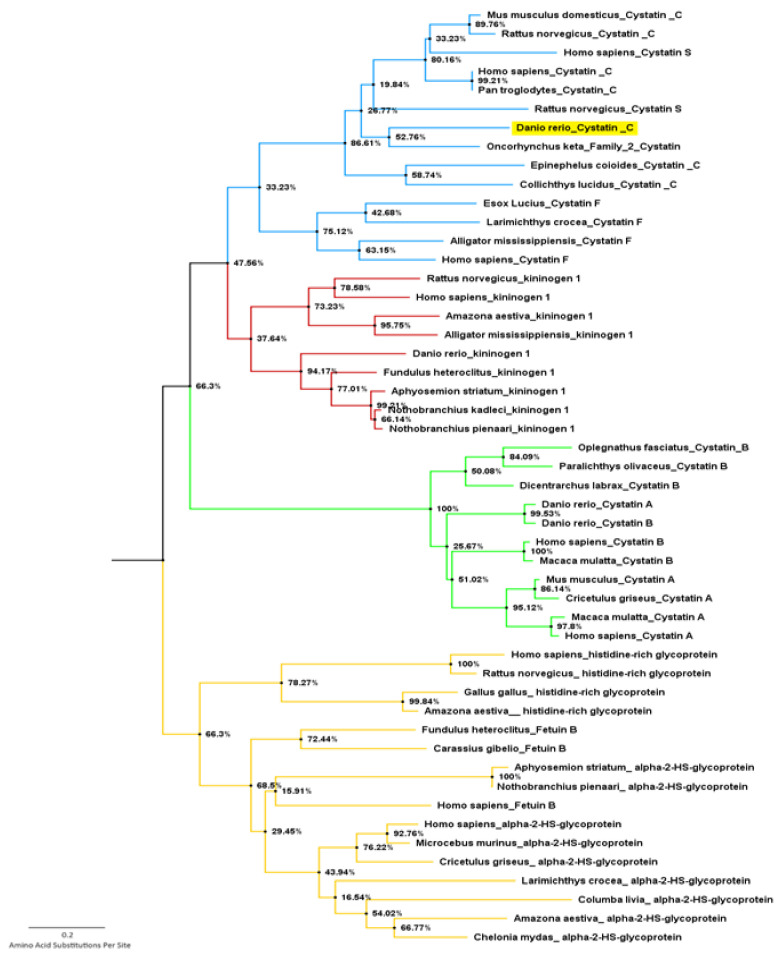
Refined phylogenetic tree. Type 1 cystatins in green, type 2 cystatins in blue, type 3 cystatins in red, and type 4 cystatins in orange. The *Danio rerio* amino sequence is highlighted in yellow.

The resulting phylogenetic tree Figure 2, obtained after iterative refinement of the complete tree (Supplementary Figure S1) revealed that the query sequence was clustered among type 2 cystatin C proteins, closely resembling the type 2 cystatin protein produced by Oncorhynchus keta, as shown in Figure 2.

In the AChE example, it could be observed that the proteins were grouped based on the phyla of Mammalia, Aves, Reptilia, and Pisces. The AChE of humans and rats was shown to share a common origin. The overall phylogenetic tree was sound, with confidence levels of over 80% (Supplementary Figure S5).

### 3.3. Identification of Conserved Sequence Motifs

**Figure 3:**
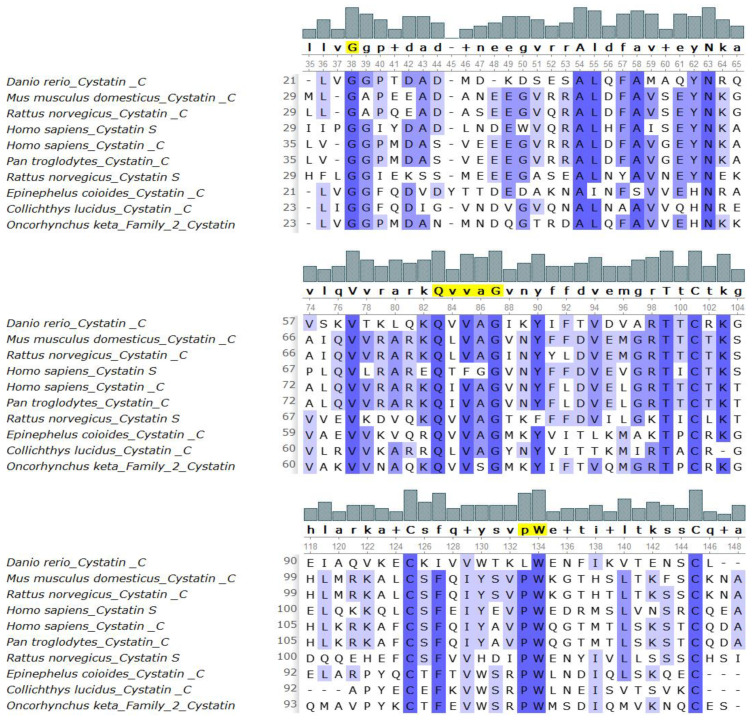
Alignment of the *Danio rerio* amino acid sequence with the closely related sequences. The conserved G, QVVAG, and P.W. motifs are highlighted in yellow. Highly conserved, strong homologies are shaded in dark blue, and weak similarities are shaded in light blue.

The cystatin C protein produced by *D. rerio* possessed three evolutionarily conserved sequence motifs: the N-terminal glycine, the QVVAG motif, and the PW motif [49]. *D. rerio* is also recognized as containing a mutation in the PW motif, which is instead read as the LW motif, as shown in Figure 3. 

In contrast, the AChE sequences for the mammalian clade had a high degree of conservation in sequence motifs. The MSA also showed the high degree of similarity between the AChE of *Rattus norvegicus* and *Homo sapiens* (Supplementary Figure S6).

### 3.4. Secondary Structure Prediction

The JPred server stated that the amino acid sequence under study coded for one short and one long alpha-helix, complete with five antiparallel beta-sheets situated between the two helix structures (Supplementary Figure S2). This complied with the typical cystatin C protein [50]. Both AChE amino acid chains had 14 alpha-helices and 12 beta-sheets. These data were in agreement with previous work conducted on AChE secondary structure prediction [13].

### 3.5. Protein Structure Prediction and Refinement

The structure predicted by I-TASSER had five antiparallel beta-sheets lying across a short and a long alpha-helix complete with two disulfide bridges. In contrast, the structure predicted *ab initio* was devoid of both the short alpha-helix and the disulfide bridges. Therefore, the homology model was selected while the latter was rejected.

**Figure 4:**
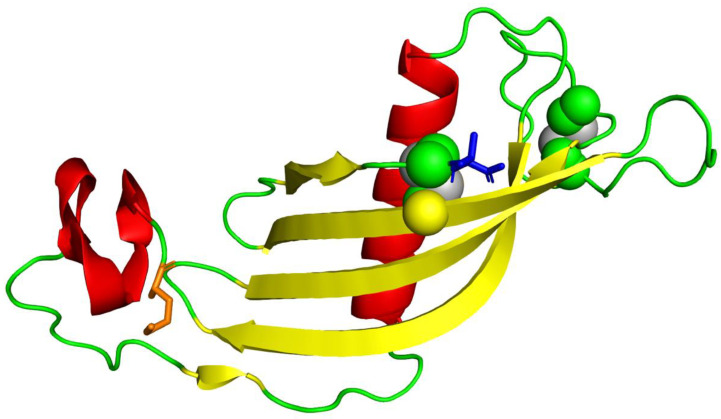
Refined tertiary structure model produced by ReFOLD server. The N-terminus (protein start) is colored in orange, the C-terminus (protein end) in blue, beta-sheets in yellow, alpha helices in red, and disulfide bonds in gray.

Quality assessment of the homology-predicted structure through the ProSA-web service resulted in a Z-score of −4.55, nested within other proteins of similar size (Supplementary Figure S3). In contrast, the local model quality assessment revealed a series of N-terminal amino acids with unfavorable, elevated knowledge-based energies. Stereochemical analysis of the model using a Ramachandran plot revealed only 67.5% of the amino acid residues occupying the most favorable region (Supplementary Figure S4). Due to the inadequacies in the structure revealed through these tests, it was decided to subject the homology-predicted model to structure refinement using the ReFOLD server. The ReFOLD server produced a high-resolution structure of 1.5 Å complete with the five antiparallel beta-sheets, a short and a long alpha-helix, and the two disulfide bridges characteristic of all cystatin C proteins. The refined structure is depicted in Figure 4.

**Figure 5:**
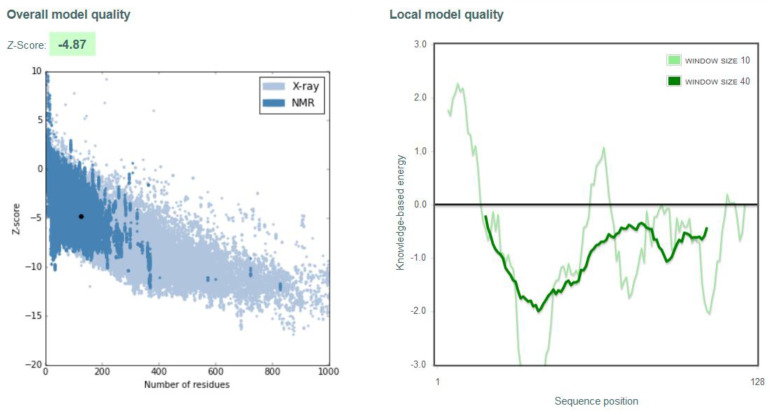
ProSA-web service quality assessment plots for refined ReFOLD model.

**Figure 6:**
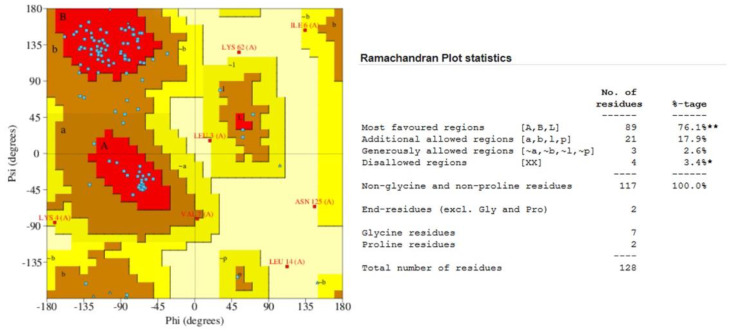
Validation of the ReFOLD-predicted protein structure using the Ramachandran plot. * *p* < 0.05, ** *p* < 0.01.

The refined structure had a confidence score of 6.31 × 10^−8^, translating to an accuracy level of 99.99%. The ReFOLD server scored the structure with a satisfactory global model quality score of 0.6671, stating that an overall improvement of 0.3% was obtained for the final structure. Quality assessment of the refined structure using the ProSA-web service revealed an improved Z-score of −4.87, including a significant reduction in the knowledge-based energies of the N-terminal residues into favorable values. The final stereochemical analysis of the refined structure via the Ramachandran plot revealed that 76.1% of the amino acid residues occupied the most favorable regions. In comparison, only 3.4% or four residues occupied the disallowed regions of the plot. The results of the ProSA-web service and the Ramachandran plot of the refined structure are shown in Figure 5 and Figure 6. 

The initially predicted AChE molecules showed certain discrepancies when subjected to the Ramachandran plot and ProSA-web server analyses refined in the same manner using the ReFOLD server, resulting in more stereochemically sound models (Supplementary Figure S7).

### 3.6. Comparative Analysis of Predicted Structure with Existing Structures

The prediction of the hAChE structure was conducted to analyze the degree of accuracy with which the predicted protein matched the existing model of the hAChE protein. The two structures did not fit perfectly, and there was some degree of variation. The RMSD of the deviation between the two structures was 0.609 Å.

### 3.7. Placement of Evolutionarily Conserved Sequence Motifs

**Figure 7:**
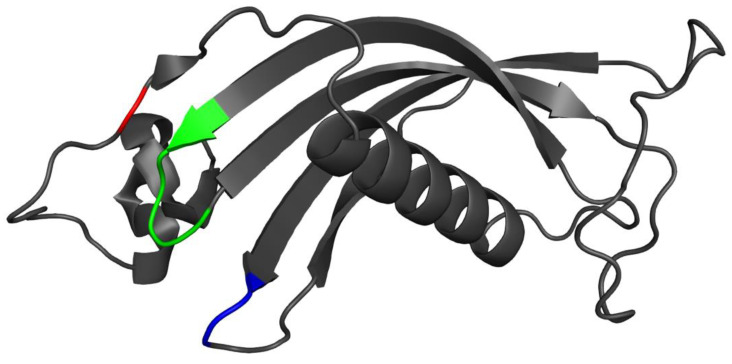
The tertiary structure of cystatin C of *Danio rerio* in gray with the N-terminal glycine residue in red, the QVVAG motif in green, and the PW motif in blue.

The amino acid sequences encoding cystatin C proteins contain three prominent evolutionarily conserved sequence motifs: the N-terminal glycine, the QVVAG motif, and a PW motif. The positioning of these sequences on the protein’s structure is directly related to the functional effectiveness [51]. The N-terminal glycine occupied the protein chain while the QVVAG motif and the PW motif inhabited the beta-hairpin turns (Figure 7). This structural placement proved that the predicted structure of the cystatin C protein produced by *D. rerio* was functionally active [52,53].

### 3.8. Surface Analysis of the Tertiary Structure

Analysis of the protein’s surface for potential active sites revealed a total of 28 regions capable of forming viable protein–protein interactions (Supplementary Table S1). Twenty regions consisted of electrostatically charged patches resulting from 6 positively charged regions and 14 negatively charged regions. The remaining 8 regions were naturally hydrophobic. The hAChE was shown to possess 109 regions capable of protein–ligand interactions with 34 positive interactions, 52 negative interactions, and 21 hydrophobic interactions. In comparison, the rAChE structure contained 141 regions in total, with 54 positive, 62 negative, and 23 hydrophobic interactions.

### 3.9. Functional Analysis via Virtual Screening

**Figure 8:**
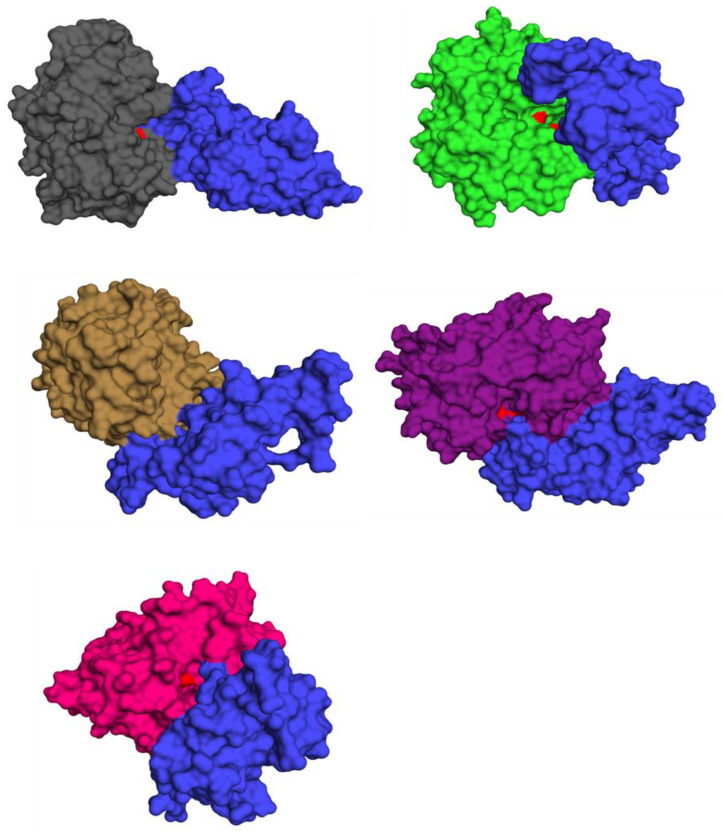
Structures of cystatin C (blue)–cysteine protease complexes with clear blocking of the protease active sites (red). Papain depicted in gray, cystatin B in green, cystatin H in brown, cystatin L1 in purple, and cystatin S in pink.

The ability of the predicted cystatin C structure to inhibit the activity of a cysteine protease enzyme was evaluated via rigid-body protein–protein docking. The cystatin C protein was first evaluated for inhibiting the cysteine protease papain (PDB ID: 9PAP). A stable enzyme–substrate complex was formed with a binding energy of −347.6 kJ/mol and the complete blockage of the ^25^Cys and ^159^His active papain sites [54]. The inhibition of cathepsins revealed promising results. Cathepsin B’s active sites of ^29^Cys, ^110^His, and ^111^His were successfully inhibited with a binding energy of −542.3 kJ/mol [55]. Cathepsin H activity was inhibited with a binding energy of −601.8 kJ/mol, with complete blockage of the ^25^Cys and ^159^His active sites [56]. Cathepsin L1 had the most robust protein-inhibitor complex, with a binding energy of −793.4 kJ/mol. The cathepsin L1 active sites of ^25^Cys and ^163^His showed complete blocking [57]. Finally, the activity of cathepsin S was inhibited with a binding energy of −435.7 kJ/mol and complete blocking of the active ^25^Cys site [58]. The resultant protein–protein complexes produced by the virtual screening exercise are depicted in Figure 8, and their detailed surface interactions are shown in Supplementary Tables S2–S6. The predictions from this analysis are novel and, therefore, will require further experimentally based analyses to confirm the results.

### 3.10. Virtual Screening Analysis for Protein–Ligand Interactions

The protein–ligand interactions were predicted using AutoDock Vina. The predicted structures of rAChE and hAChE were subjected to virtual screening with echothiophate. The purpose of using two different types of software was to enable us to identify whether both programs would produce the same result.

**Figure 9:**
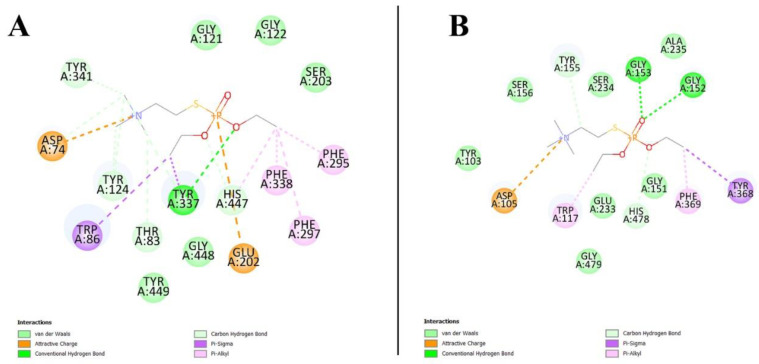
A 2D representation of the protein–ligand complex formed in the active-site gorge between AChE and echothiophate. (**A**) The interactions in hAChE with strong interactions between the ligand and the characteristic tryptophan residue. (**B**) The interactions in rAChE showing tryptophan residues involved in forming the stable protein–ligand complex.

The active site of AChE is situated in a centrally placed gorge [59]. The active-site gorge contains a tryptophan residue involved in the formation of protein–ligand interactions [13]. Coupled with this information, AutoDock Vina predicted the binding of the echothiophate ligand to the active site in both of the rAChE and hAChE macromolecules. The binding affinity was −5.6 kcal/mol for both proteins (Supplementary Tables S7 and S8). In contrast, the negative control showed weak binding to both proteins: −3.2 kcal/mol for rAChE and −3.3 kcal/mol for hAChE (Supplementary Tables S9 and S10). However, these complexes did not occupy the active-site gorge, thus confirming that the binding of the echothiophate is not random (Figure 9). This also provides evidence that the echothiophate ligand is a potential competitive inhibitor of AChE proteins. Further experimental analysis will be required to confirm these predictions.

Through our replication studies, we were further able to validate the use of our pipeline and its selected tools in obtaining a comprehensive understanding of a protein’s function. The first analysis replicated the protein–ligand interaction between the survivin protein and 4-hydroxy pyridine 1-oxide pyridin-4-ol 1-oxide ligand. The original authors had conducted a series of tests to identify the active compounds, such as the 4-hydroxy pyridine 1-oxide pyridin-4-ol 1-oxide present in *Nyctanthes arbor-tristis*. These have proven their anticancer properties through anticancer assays involving cell cultures. Additionally, these authors investigated the interaction of the aforementioned active compound with survivin to identify its anticancer mechanism [15]. We replicated this portion of the analysis using AutoDock Vina and conducted our analysis of the binding site. AutoDock Vina’s predictions shared a significant number of similarities with those of the original work, including the involved residues and their interactions, for example, the pi-cation interaction formed between the ligand and ^18^Arg (Supplementary Figure S8). The binding affinities were near-identical, with ours at −4.6 kcal/mol and that of the original authors at −5.449 kcal/mol (Supplementary Table S11).

Our second replication study was based on the X-ray crystallography analysis of hemoglobin and the haem complex formed in the presence of oxygen [16]. Our analysis revealed favorable binding with an affinity of −11.4 kcal/mol (Supplementary Table S12). The haem ligand was found to be nestled inside the pocket of the protein. Additionally, the characteristic “heme coordinated to the histidine residue” protein–ligand interaction was present between the haem’s central iron atom and the hemoglobin’s histidine amino acid (Supplementary Figure S9) [60]. 

### 3.11. Prediction of Cystatin C Active Binding Site

**Figure 10:**
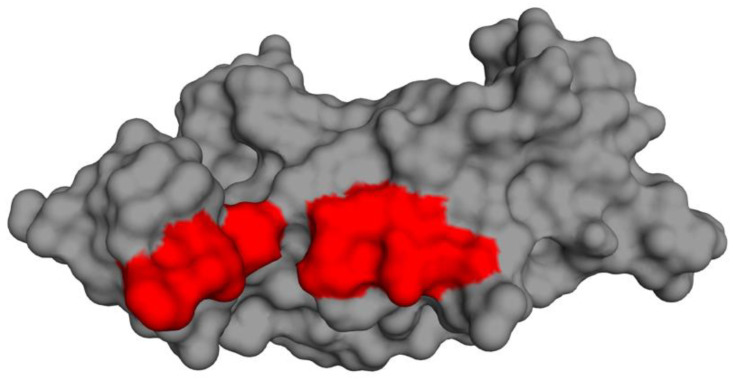
Surface view of the protein’s tertiary structure (gray) with the active binding site marked in red.

Statistical analysis of the interacting residues of the cystatin C protein responsible for the inhibition of cysteine protease activity revealed seven closely situated amino acids. These residues were identified as ^2^Phe, ^3^Leu, ^9^Phe, ^124^Glu, ^125^Asn, ^126^Ser, and ^127^Cyx. The seven residues displayed their activity in the inhibition of the cathepsin proteins. In all instances, these residues were present in the formation of the enzyme–substrate complex that led to the inhibition of the cathepsin active site by the cystatin C protein. The predicted active binding site based on these findings is depicted in Figure 10.

### 3.12. Human Gene Interaction Mapping of Cathepsin Pathways

**Figure 11:**
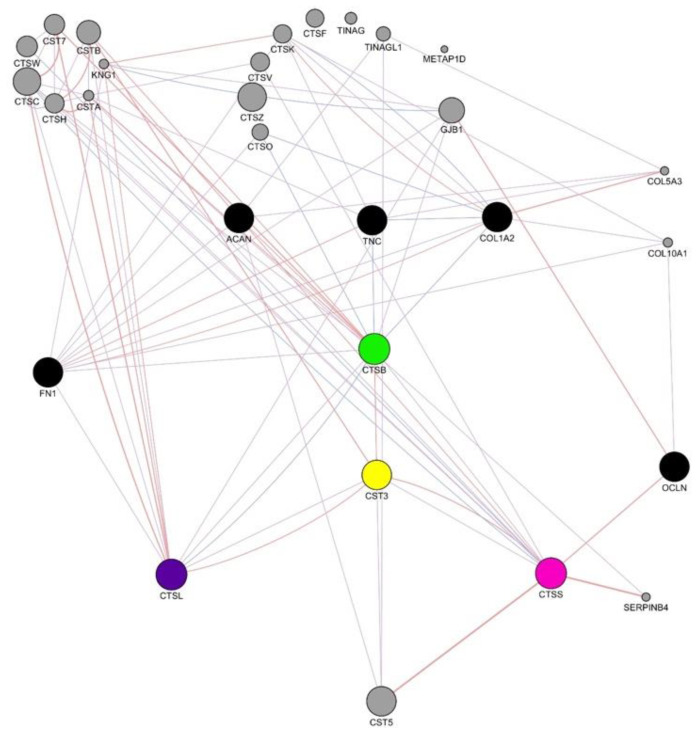
Human gene interaction network of cathepsin B (green), cathepsin L1 (purple), and cathepsin S (pink). Clinically significant gene interactions are shown in black.

Gene interaction mapping of cathepsin B, cathepsin L1, and cathepsin S revealed a series of clinically significant pathways where cathepsin overexpression would lead to protein degradation and subsequent illness. The results of the gene interaction mapping are depicted in Figure 11.

Cathepsin B overexpression results in the onset of osteoporosis, rheumatoid arthritis, and certain forms of cancers due to the excessive degradation of the proteins aggrecan, tenascin C, fibronectin, and collagen type 1 [57]. Rigid-body protein docking of aggrecan (PDB ID: 4M4D), tenascin C (PDB ID: 2RB8), fibronectin (PDB ID:1E8B), and collagen type 1 (PDB ID: 3EJH) with cathepsin B revealed successful enzyme–substrate complexes with binding energies of −263.2 kJ/mol, −500.1 kJ/mol, −551.8 kJ/mol, and −79.5 kJ/mol, respectively. All four of these binding energies except that of fibronectin were greater than that of cystatin C of *D. rerio* and cathepsin B, at −549.2 kJ/mol.

Cathepsin L1 overexpression has been proven to lead to the degradation of fibronectin, resulting in the onset of melanomas [61]. Cathepsin L1 formed an enzyme–substrate complex with fibronectin with a binding energy of −508.7 kJ/mol, which was greater than the binding energy of the cystatin C protein under study and the cathepsin L1 protein, at −793.4 kJ/mol.

Visualization of the gene interaction network of cathepsin S revealed the degradation of occludin, which would result in the metastasis of cancer cells causing bone and breast cancers [62]. Protein docking of cathepsin S with occludin resulted in a low binding energy of −488.5 kJ/mol, which was lower than that of the cystatin C and cathepsin S complex, at −454.4 kJ/mol.

## 4. Discussion

The primary objective of the present study was to design a novel procedure that attempts to identify, characterize, and predict the structure of a protein under study, prove its functional capability and predict its therapeutic potential for the treatment of diseases. Although this is the standard procedure for protein analysis, a complete single study encompassing these steps in order with benchmarked tools was lacking. In the present study, we produced such a protocol to validate the importance of each step in the process of protein analysis. We corroborated the selection of our tools through replication studies and literature-based reviews. Finally, we showed the robustness of our protocol and its information-rich results through three novel studies unique to this work, namely, the study of the cystatin C protein of *D. rerio* and the interactions of the rAChE and hAChE proteins with echothiophate.

Proteins belonging to the same family usually have a series of conserved sequence motifs that act as a fingerprint, enabling the classification of novel sequences. The cystatin C coding amino acid sequence of *Danio rerio* obtained from the NCBI database contained one cystatin coding domain consisting of an N-terminal glycine and a QxVxG sequence motif [63]. The conserved domain search service revealed the presence of a cystatin domain with two evolutionarily conserved sequence motifs. If a DNA sequence coding of a novel protein is present, it is recommended to commence the study using NCBI’s blastx search [64].

Conserved segments in DNA or protein sequences play a role in the function of the macromolecules encoded by them [65]. Likewise, proteins with similar functions belong to the same family. Therefore, positioning a predicted protein among others with shared functions and the presence of these evolutionarily conserved sequences enable the validation of a predicted protein [66].

The phylogenetic construction of the cystatin superfamily tree conducted by the MEGA 6 software revealed a cystatin C dendrogram that coincided with the existing validated cystatin protein family. The generated phylogenetic tree depicted in Figure 2 showed a common ancestry between the type 1 cystatins, cystatin A and B, which branch off to a shared ancestry between type 2 and type 3 cystatins, with a completely separate branching shown by type 4 cystatins or fetuins. This phylogenetic pattern was successfully reproduced in the study and is characteristic of the cystatin superfamily [10]. The protein considered in this study was found to be positioned among the type 2 cystatin C proteins, validating the phylogenetic tree and confirming its accurate prediction.

Executing a multiple sequence alignment (MSA) post phylogenetic analysis enables the accurate recognition of evolutionarily conserved sequence motifs among closely related proteins involved in the protein’s characteristic functions. It also allows identification of the sequence motifs that are most critical in the governance of the protein’s function [67]. Cystatin C proteins are thought to have three such evolutionarily preserved sequence motifs, namely, an N-terminal glycine, a QVVAG motif, and a PW motif [10]. The MSA revealed the presence of all three sequence motifs, conserved in the polypeptide chain of the protein under study.

The next step after protein characterization is the functional analysis, which requires predicting the protein’s native 3D tertiary structure. This involves the identification of the proteins’ secondary structure first [2]. The secondary structure of a protein determines the positioning of the alpha-helices and beta-sheets in the final 3D structure. In proteins such as cystatin C, a specific configuration of secondary structures is vital for its function. A typical cystatin C protein’s secondary structure must have two alpha-helices, i.e., one short and one long alpha-helix, and between them, five antiparallel beta-sheets [68]. These features were shown to be present by the JPred server in the amino acid sequence under study.

The prediction of a protein’s tertiary structure through computational techniques has a two-fold advantage. The conventional methods of NMR and X-ray crystallography are expensive and not readily available to everyone. In addition, the structure of short-half-life proteins cannot be predicted using these methods [1]. The current pipeline successfully utilizes the two main protein modeling methods, namely homology and *ab initio* modeling [69].

The I-TASSER server was utilized for the homology prediction of the protein structure. The I-TASSER server uses a hierarchical approach to predicting protein structures [26]. Its reliability has been proven by critical assessment of protein structure prediction (CASP) experiments [70]. The predicted structure contained all the structural components that must be present in a cystatin C protein, such as both a short and a long alpha-helix, five antiparallel beta-sheets, and two disulfide bonds [50].

*Ab initio* or *de novo* protein modeling was conducted utilizing the QUARK server. In the absence of available templates, this technique can predict the protein structure from the amino acid sequence alone [69]. The QUARK server was selected due to its reliability, proven by CASP experiments [28]. However, the *ab initio* predicted model failed the validity test, since both of its alpha-helices were long and there were no disulfide bonds.

The structure of a protein depends on its sequence. A proper tertiary structure should have minimum free energy and be stereochemically stable [71]. These two tests were carried out by the ProSA-web test, and the Ramachandran plots revealed that the structure had a few discrepancies. In this event, the refinement of the protein has proven to be an efficient solution. The ReFOLD server validated through the CASP12 experiments could remove these discrepancies, to show significant improvements in retesting the refined model [40].

It is known that electrostatically charged and hydrophobic residues on a protein’s surface play a key role in long-distance protein–protein interactions and are vital for drug design. Therefore these regions were mapped before conducting the virtual screening process [72]. The cystatin C under study had 35 such regions.

Cystatin C proteins are thought to be the most active protease inhibitors in the entire cystatin family. They can competitively block the active sites of papain and cathepsins B, H, L, and S [10]. Protein–protein docking proved this statement to be accurate, as in each instance, the cysteine active site of the cathepsin protein was blocked by the cystatin C protein. Based on the Gibbs free energy laws, proteins forming valid complexes should have negative values. The more negative the value, the more stable the complex [1]. The inhibition of all the cathepsins occurred under favorable negative binding energies, further confirming the results of the pipeline’s chosen docking software.

Residues that are frequently involved in protein–protein complexes are believed to be related to the function of the protein [4]. Such residues existed for the active protein site [73]. Based on the above principle, the cystatin C protein’s active site was identified by recognizing the amino acid sequences that were always involved in inhibiting the cathepsin proteins (Figure 5).

Genetic interaction mapping reveals functional pathways involved in molecular mechanisms [74]. Some of these mechanisms can become clinically significant if compromised and can lead to disease conditions such as tumorigenesis. Therefore, the study of gene–gene interaction maps of model organisms and humans has been incorporated into drug discovery and design [75]. Gene interaction mapping of cathepsins B, L1, and S revealed several clinically compromised pathways caused by cathepsin overexpression (Figure 11). Since the cystatin C protein is a competitive inhibitor, cross-analysis of the binding affinity of cathepsins with their natural substrates and the cystatin C protein should reveal the potential of the protein under study to act as a potential drug [76]. Viable candidates were diseases caused by overexpression of cathepsins B and L1.

Benchmarking the pathway was possible using the prediction of the structure and function of the cystatin C of *D. rerio*. To further test the reliability and accuracy of the proposed pathway and the software used, the AChE proteins of both humans and rats were selected, enabling the extension of the pathway from protein–protein to protein–ligand analysis.

The hAChE macromolecule was used to test the reliability of the I-TASSER software against already identified protein structures. The hAChE protein’s tertiary structure already exists in the PDB data bank. Even though the server predicted a structure closely resembling the accurate native model after refinement, minor deviations resulted in an RMSD with a value of 0.609 Å. Apart from this, both models were successfully analyzed for their functionality.

The AChE protein was utilized to comparatively analyze the use of AutoDock Vina. When the grid box was implemented accurately to accommodate the respective active sites, AutoDock Vina was able to bind the ligands accurately. When faced with a false negative, the ligand bound away from the active site, showing that Vina’s predictions were not random.

Th proposed pipeline, therefore, ensures the complete characterization of the protein under study. The final result confirms that the user can be provided with the function of the macromolecule and an analysis complete with the query sequence’s evolutionary significance, structural characteristics, and the prediction of tertiary structures that are appropriately validated and refined. The pipeline is robust enough to identify putative active sites also.

## 5. Conclusions

In conclusion, the proposed computational pipeline utilizing only free, open-source software can be used to conduct a complete analysis of a novel amino acid sequence, revealing its identity and its functional and therapeutic potential. Using the cystatin C and AChE proteins and a series of replication studies, we were able to prove the proposed pipeline’s robustness. The pipeline characterized the protein based on its evolutionary relatedness, overcame protein modeling errors through structure refinement, and established the protein’s functionality complete with its active site and therapeutic potential. Such analysis has become crucial in developing targeted therapeutics intended to inhibit a protein’s active site. Following the successes of genome sequencing, novel proteins’ open reading frames (ORFs) are being discovered daily, and with the success of protein structure prediction techniques such as AlphaFold 2, the number of known structures is gradually increasing. Therefore, we believe that this single, economic pipeline will assist in bridging the gap between known protein sequences and those that are experimentally validated. Future work will focus on using our methodology to conduct trifecta analyses on a large scale and on further refinement of the protocol to enable expansion into large datasets.

## Data Availability

All the data and software utilized in the design and benchmarking of the pipeline are open source and freely available for download.

## Supplementary Materials

The following are available online at https://www.mdpi.com/article/10.3390/biology10111113/s1, Figure S1: Complete initial phylogenetic tree. Figure S2: JPred secondary structure prediction server re-sult. The ‘H’ represents a helix, ‘E’ represents a beta sheet. Figure S3: ProSA-web service quality assessment plots for the I-Tasser generated model before refinement. Figure S4: Validation of the I-TASSER predicted protein structure using the Ramachandran plot before refinement. Figure S5: Phylogenetic Tree Analysis of Acetylcholinesterase. Clear distinct clade separation represented as follows with Mammalian Ache in Red, Aves Ache in Green, Pisces in Blue and Invertebrate Ache in Purple. Homo sapiens and Rattus norvegicus highlighted in yellow. Figure S6: Multiple Se-quence Alignment by ClustalW of mammalian Ache. Symbol * depicts highly conserved sequence motifs. Homo sapiens sequence highlighted in yellow and Rattus norvegicus highlighted in green. Figure S7: The three-dimensional alignment of the AChE crystal structure of Rattus norvegicus (red) against the AChE crystal structure of Homo sapiens (blue). Figure S8: The 2D representation of the protein-ligand complex formed in the Survivin protein and 4-hydroxypyridine 1-oxide pyr-idin-4-ol 1-oxide. The amin acid residues that were present in the original study have been marked by a red underline. Figure S9: The 2D representation of the protein-ligand complex formed in the Hemoglobin and the heam ligand. The characteristic “heme coordinated to the his-tidine residue” protein-ligand interaction can be seen between the heam ligand’s central iron atom and the Haemoglobin’s Histidine amino acid. Table S1: Detailed description of the electrostatic charge and hydrophobic residue distribution on the protein surface of Cystatin C of D. rerio, Table S2: Protein Surface Analysis of the interacting surfaces between Cystatin C (A) and Papain (B). Details regarding the types of bonding including Hydrogen Bonding (HB), Salt Bridges (SB), Pi Stacking (Pi), Disulfide bonding (DS) and Vander Waal interactions (VW). Table S3: Protein Sur-face Analysis of interacting surfaces between Cystatin C (A), Cathepsin B (B). Details regarding the types of bonding including Hydrogen Bonding (HB), Salt Bridges (SB), Pi Stacking (Pi), Disul-fide bonding (DS) and Vander Waal interactions (VW). Table S4: Protein Surface Analysis of in-teracting surfaces between Cystatin C (A), Cathepsin H (B). Details regarding the types of bond-ing including Hydrogen Bonding (HB), Salt Bridges (SB), Pi Stacking (Pi), Disulfide bonding (DS) and Vander Waal interactions (VW). Table S5: Protein Surface Analysis of interacting surfaces between Cystatin C (A), Cathepsin L1 (B). Details regarding the types of bonding including Hy-drogen Bonding (HB), Salt Bridges (SB), Pi Stacking (Pi), Disulfide bonding (DS) and Vander Waal interactions (VW). Table S6: Protein Surface Analysis of interacting surfaces between Cystatin C (A), Cathepsin S (B). Details regarding the types of bonding including Hydrogen Bonding (HB), Salt Bridges (SB), Pi Stacking (Pi), Disulfide bonding (DS) and Vander Waal interactions (VW). Table S7: AutoDock Vina results for protein ligand docking between hAChE and Echothiophate. Table S8: AutoDock Vina results for protein ligand docking between rAChE and Echothiophate. Table S9: AutoDock Vina results for protein ligand docking between hAChE and the negative control Imidazole. Table S10: AutoDock Vina results for protein ligand docking between rAChE and the negative control Imidazole. Table S11: AutoDock Vina results for protein ligand docking between Survivin protein and 4-hydroxypyridine 1-oxide pyridin-4-ol 1-oxide. Table S12: Auto-Dock Vina results for protein ligand docking between Haemoglobin and Heam.

## References

[1] Zarbafian S., Moghadasi M., Roshandelpoor A., Nan F., Li K., Vakli P., Vajda S., Kozakov D., Paschalidis I.C. (2018). Protein docking refinement by convex underestimation in the low-dimensional subspace of encounter complexes. Sci. Rep..

[2] Godbey W. (2014). Proteins. Introd. Biotechnol..

[3] Skarzyńska A., Pawełkowicz M., Krzywkowski T., Świerkula K., Pląder W., Przybecki Z. (2015). Bioinformatics pipeline for functional identification and characterization of proteins. Photonics Appl. Astron. Commun. Ind. High-Energy Phys. Exp..

[4] Bertoni M., Kiefer F., Biasini M., Bordoli L., Schwede T. (2017). Modeling protein quaternary structure of homo- and hetero-oligomers beyond binary interactions by homology. Sci. Rep..

[5] Yang J., Yan R., Roy A., Xu D., Poisson J., Zhang Y. (2015). The I-TASSER Suite: Protein structure and function prediction. Nat. Methods.

[6] Margulies E.H., Blanchette M., Program N.C.S., Haussler D., Green E.D. (2003). Identification and characterization of multi-species conserved sequences. Genome Res..

[7] Wong A., Gehring C., Irving H.R. (2015). Conserved functional motifs and homology modeling to predict hidden moonlighting functional sites. Front. Bioeng. Biotechnol..

[8] Pruess M., Apweiler R. (2003). Bioinformatics resources for in silico proteome analysis. J. Biomed. Biotechnol..

[9] Clark K., Balciunas D., Pogoda H.-M., Ding Y., Westcot S.E., Bedell V., Greenwood T.M., Urban M.D., Skuster K.J., Petzold A. (2011). In vivo protein trapping produces a functional expression codex of the vertebrate proteome. Nat. Methods.

[10] Ochieng J., Chaudhuri G. (2010). Cystatin superfamily. J. Health Care Poor Underserved.

[11] Magister Š., Kos J. (2013). Cystatins in immune system. J. Cancer.

[12] Chen Y.Z. (2002). TTD: Therapeutic target database. Nucleic Acids Res..

[13] Dvir H., Silman I., Harel M., Rosenberry T.L., Sussman J.L. (2010). Acetylcholinesterase: From 3D structure to function. Chem. Interact..

[14] Dym O., Unger T., Toker L., Silman I., Sussman J., Center I.S.P. (2014). Crystal structure of human acetylcholinesterase. Isr. Struct. Proteom. Cent..

[15] Heendeniya S.N., Keerthirathna L., Manawadu C.K., Dissanayake I.H., Ali R., Mashhour A., Alzahrani H., Godakumbura P., Boudjelal M., Peiris D.C. (2020). Therapeutic efficacy of Nyctanthes arbor-tristis flowers to inhibit proliferation of acute and chronic primary human leukemia cells, with adipocyte differentiation and in silico analysis of interactions between survivin protein and selected secondary metabolites. Biomolecules.

[16] Paoli M., Liddington R., Tame J., Wilkinson A., Dodson G. (1996). Crystal structure of T state haemoglobin with oxygen bound at all four haems. J. Mol. Biol..

[17] Lu S., Wang J., Chitsaz F., Derbyshire M.K., Geer R.C., Gonzales N.R., Gwadz M., Hurwitz D.I., Marchler G.H., Song J.S. (2020). CDD/SPARCLE: The conserved domain database in 2020. Nucleic Acids Res..

[18] Pruitt K. (2003). NCBI reference sequence project: Update and current status. Nucleic Acids Res..

[19] Marchler-Bauer A., Derbyshire M.K., Gonzales N.R., Lu S., Chitsaz F., Geer L.Y., Geer R.C., He J., Gwadz M., Hurwitz D.I. (2015). CDD: NCBI’s conserved domain database. Nucleic Acids Res..

[20] Barrett T., Troup D.B., Wilhite S.E., LeDoux P., Evangelista C., Kim I.F., Tomashevsky M., Marshall K.A., Phillippy K.H., Sherman P.M. (2010). NCBI GEO: Archive for functional genomics data sets—10 years on. Nucleic Acids Res..

[21] Geer L.Y., Marchler-Bauer A., Geer R.C., Han L., He J., He S., Liu C., Shi W., Bryant S.H. (2009). The NCBI BioSystems database. Nucleic Acids Res..

[22] Cock P.J.A., Chilton J., Grüning B., Johnson J.E., Soranzo N. (2015). NCBI BLAST+ integrated into Galaxy. GigaScience.

[23] Tamura K., Stecher G., Peterson D., Filipski A., Kumar S. (2013). MEGA6: Molecular evolutionary genetics analysis version 6.0. Mol. Biol. Evol..

[24] Tamura K., Stecher G., Kumar S. (2021). MEGA11: Molecular evolutionary genetics analysis version 11. Mol. Biol. Evol..

[25] Yang Z. (2007). PAML 4: Phylogenetic analysis by maximum likelihood. Mol. Biol. Evol..

[26] Roy A., Kucukural A., Zhang Y. (2010). I-TASSER: A unified platform for automated protein structure and function prediction. Nat. Protoc..

[27] Xu D., Zhang Y. (2012). Ab initio protein structure assembly using continuous structure fragments and optimized knowledge-based force field. Proteins Struct. Funct. Bioinform..

[28] Zhang W., Yang J., He B., Walker S.E., Zhang H., Govindarajoo B., Virtanen J., Xue Z., Shen H.-B., Zhang Y. (2016). Integration of QUARK and I-TASSER for Ab Initio Protein Structure Prediction in CASP11. Proteins Struct. Funct. Bioinform..

[29] Jumper J., Evans R., Pritzel A., Green T., Figurnov M., Ronneberger O., Tunyasuvunakool K., Bates R., Žídek A., Potapenko A. (2021). Highly accurate protein structure prediction with AlphaFold. Nat. Cell Biol..

[30] Ritchie D.W., Venkatraman V. (2010). Ultra-fast FFT protein docking on graphics processors. Bioinformatics.

[31] Ghoorah A.W., Devignes M.-D., Smaïl-Tabbone M., Ritchie D.W. (2013). Protein docking using case-based reasoning. Proteins Struct. Funct. Bioinform..

[32] Trott O., Olson A.J. (2010). AutoDock Vina: Improving the speed and accuracy of docking with a new scoring function, efficient optimization, and multithreading. J. Comput. Chem..

[33] MacIndoe G., Mavridis L., Venkatraman V., Devignes M.-D., Ritchie D.W. (2010). HexServer: An FFT-based protein docking server powered by graphics processors. Nucleic Acids Res..

[34] Agrawal P., Singh H., Srivastava H.K., Singh S., Kishore G., Raghava G.P.S. (2019). Benchmarking of different molecular docking methods for protein-peptide docking. BMC Bioinform..

[35] Okonechnikov K., Golosova O., Fursov M., The UGENE Team (2012). Unipro UGENE: A unified bioinformatics toolkit. Bioinformatics.

[36] Drozdetskiy A., Cole C., Procter J., Barton G.J. (2015). JPred4: A protein secondary structure prediction server. Nucleic Acids Res..

[37] DeLano W.L. (2002). PyMOL: An open-source molecular graphics tool. CCP4 Newsl. Protein Crystallogr..

[38] Wiederstein M., Sippl M.J. (2007). ProSA-web: Interactive web service for the recognition of errors in three-dimensional structures of proteins. Nucleic Acids Res..

[39] Laskowski R.A., Macarthur M.W., Thornton J.M. (2001). PROCHECK: Validation of protein-structure coordinates. Int. Tables Crystallogr..

[40] Shuid A.N., Kempster R., McGuffin L.J. (2017). ReFOLD: A server for the refinement of 3D protein models guided by accurate quality estimates. Nucleic Acids Res..

[41] Zhu K., Day T., Warshaviak D., Murrett C., Friesner R., Pearlman D. (2014). Antibody structure determination using a combination of homology modeling, energy-based refinement, and loop prediction. Proteins Struct. Funct. Bioinform..

[42] Hwang H., Vreven T., Janin J., Weng Z. (2010). Protein-protein docking benchmark version 4.0. Proteins Struct. Funct. Bioinform..

[43] Ritchie D.W. (2003). Evaluation of protein docking predictions usingHex 3.1 in CAPRI rounds 1 and 2. Proteins Struct. Funct. Bioinform..

[44] Laskowski R.A., Swindells M.B. (2011). LigPlot+: Multiple ligand-protein interaction diagrams for drug discovery. ACS Pub..

[45] Patel M., Patel L.J. (2014). Design, synthesis, molecular docking, and antibacterial evaluation of some novel flouroquinolone derivatives as potent antibacterial agent. Sci. World J..

[46] Kopitar-Jerala N. (2006). The role of cystatins in cells of the immune system. FEBS Lett..

[47] Warde-Farley D., Donaldson S.L., Comes O., Zuberi K., Badrawi R., Chao P., Franz M., Grouios C., Kazi F., Lopes C.T. (2010). The GeneMANIA prediction server: Biological network integration for gene prioritization and predicting gene function. Nucleic Acids Res..

[48] Holmquist M. (2000). Alpha beta-hydrolase fold enzymes structures, functions and mechanisms. Curr. Protein Pept. Sci..

[49] Paraoan L., Hiscott P., Gosden C., Grierson I. (2010). Cystatin C in macular and neuronal degenerations: Implications for mechanism(s) of age-related macular degeneration. Vis. Res..

[50] Kolodziejczyk R., Michalska K., Hernandez-Santoyo A., Wahlbom M., Grubb A., Jaskolski M. (2010). Crystal structure of human cystatin C stabilized against amyloid formation. FEBS J..

[51] Premachandra H., Wan Q., Elvitigala D.A.S., De Zoysa M., Choi C.Y., Whang I., Lee J. (2012). Genomic characterization and expression profiles upon bacterial infection of a novel cystatin B homologue from disk abalone (Haliotis discus discus). Dev. Comp. Immunol..

[52] Björk I., Brieditis I., Raub-Segall E., Pol E., Håkansson K., Abrahamson M. (1996). The importance of the second hairpin loop of cystatin C for proteinase binding. Characterization of the interaction of Trp-106 variants of the inhibitor with cysteine proteinases. Biochemistry.

[53] Lewandowska A., Ołdziej S., Liwo A., Scheraga H.A. (2010). β-hairpin-forming peptides; models of early stages of protein folding. Biophys. Chem..

[54] Fonović M., Turk B. (2014). Cysteine cathepsins and extracellular matrix degradation. Biochim. Et Biophys. Acta (BBA)-Gen. Subj..

[55] Musil D., Zucic D., Turk D., Engh R.A., Mayr I., Huber R., Popovic T., Turk V., Towatari T., Katunuma N. (1991). The refined 2.15 A X-ray crystal structure of human liver ca-thepsin B: The structural basis for its specificity. EMBO J..

[56] Gunčar G., Podobnik M., Pungerčar J., BorutŠtrukelj B., Turk V., Turk D. (1998). Crystal structure of porcine cathepsin H determined at 2.1 å resolution: Location of the mini-chain C-terminal carboxyl group defines cathepsin H aminopeptidase function. Structure.

[57] Gunčar G., Pungercic G., Klemenčič I., Turk V., Turk D. (1999). Crystal structure of MHC class II-associated p41 Ii fragment bound to cathepsin L reveals the structural basis for differentiation between cathepsins L and S. EMBO J..

[58] McGrath M.E., Palmer J.T., Brömme D., Somoza J.R. (1998). Crystal structure of human cathepsin S. Protein Sci..

[59] Axelsen P.H., Harel M., Silman I., Sussman J.L. (1994). Structure and dynamics of the active site gorge of acetylcholinesterase: Synergistic use of molecular dynamics simulation and X-ray crystallography. Protein Sci..

[60] Nye D.B., LeComte J.T.J. (2018). Replacement of the distal histidine reveals a noncanonical heme binding site in a 2-on-2 hemoglobin. Biochemistry.

[61] Chakraborti S., Chakraborti T., Dhalla N.S. (2017). Proteases in Human Diseases.

[62] Martin T.A., Jordan N., Davies E.L., Jiang W. (2016). Metastasis to bone in human cancer is associated with loss of occludin expression. Anticancer. Res..

[63] Dutt S., Singh V., Marla S.S., Kumar A. (2010). In silico analysis of sequential, structural and functional diversity of wheat cystatins and its implication in plant defense. Genom. Proteom. Bioinform..

[64] Altschul S.F., Gish W., Miller W., Myers E.W., Lipman D.J. (1990). Basic local alignment search tool. J. Mol. Biol..

[65] Stojanovic N., Florea L., Riemer C., Gumucio D., Slightom J., Goodman M., Miller W., Hardison R. (1999). Comparison of five methods for finding conserved sequences in multiple alignments of gene regulatory regions. Nucleic Acids Res..

[66] Kordiš D., Turk V. (2009). Phylogenomic analysis of the cystatin superfamily in eukaryotes and prokaryotes. BMC Evol. Biol..

[67] Jankun-Kelly T., Lindeman A.D., Bridges S.M. (2009). Exploratory visual analysis of conserved domains on multiple sequence alignments. BMC Bioinform..

[68] Abrahamson M., Alvarez-Fernandez M., Nathanson C.-M. (2003). Cystatins. Biochem. Soc. Symp..

[69] Zhang Z. (2002). An Overview of Protein Structure Prediction: From Homology to Ab Initio. https://www.semanticscholar.org/paper/An-Overview-of-Protein-Structure-Prediction-%3A-From-Zhang/522af9cf5d1c3e4c1506d449286de6d3ebbd07ef.

[70] Zhang Y., Arakaki A.K., Skolnick J. (2005). TASSER: An automated method for the prediction of protein tertiary structures in CASP6. Proteins Struct. Funct. Bioinform..

[71] Miklos A.C., Li C., Pielak G.J. (2009). Using NMR-detected backbone amide 1H exchange to assess macromolecular crowding effects on globular-protein stability. Methods Enzymol..

[72] Keskin O., Gursoy A., Ma B., Nussinov R. (2008). Principles of protein−protein interactions: What are the preferred ways for proteins to interact?. Chem. Rev..

[73] Chatterjee A., Roy U.K., Halder D. (2011). Protein Active Site Structure Prediction Strategy and Algorithm. Int. J. Curr. Eng. Technol..

[74] Jaimovich A., Rinott R., Schuldiner M., Margalit H., Friedman N. (2010). Modularity and directionality in genetic interaction maps. Bioinformatics.

[75] Saxena N., Saxena V.S.N. (2015). Gene-gene interaction mapping of human cytomegalic virus through system biology approach. Biol. Syst. Open Access.

[76] Lionta E., Spyrou G., Vassilatis D.K., Cournia Z. (2014). Structure-based virtual screening for drug discovery: Principles, applications and recent advances. Curr. Top. Med. Chem..

