# Supplementary material for: A Novel In Silico Benchmarked Pipeline Capable of Complete Protein Analysis: A Possible Tool for Potential Drug Discovery
Source: Biology (Basel). 2021 Oct 28;10(11):1113. doi: 10.3390/biology10111113 (PMC8615085; doi:10.3390/biology10111113)
Supplement: Supplementary file 1 [file biology-10-01113-s001.zip › biology-1400312-supplementary.pdf]

A Novel *In Silico* Benchmarked Pipeline Capable of Complete Protein Analysis: A Possible Tool for Potential Drug Discovery

Authors

D. D. B. D. Perera [1,*,†,‡], K.Minoli L. Perera [1] and Dinithi C. Peiris [2,*,‡]

Affiliations

1 Department of Zoology, Faculty of Applied Sciences, University of Sri Jayewardenepura,

Nugegoda 10250, Sri Lanka; mperera95826@gmail.com

2 Genetics & Molecular Biology Unit (Center for Biotechnology), Department of Zoology, Faculty of Applied Sciences, University of Sri Jayewardenepura, Nugegoda 10250, Sri Lanka

* Correspondence: desh.02236@gmail.com (D.D.B.D.P.); dinithi@sci.sjp.ac.lk (D.C.P.); Tel.: +94-714-018-537 (D.C.P.)

† Current address: Department of Biochemistry & Molecular Biology, Cumming School of Medicine, University of Calgary, Calgary, AB T2N 1N4, Canada.

‡ Equal corresponding authors.

CONTENTS

1. Analysis of Cystatin C 3

2. Analysis of Human and Rat AChE 11

3. Docking analysis between Survivin protein and 4-hydroxypyridine 1-oxide pyridin-4-ol 1-oxide 18

4. Docking analysis between Haemoglobin and Heam. 20

1. Analysis of Cystatin C

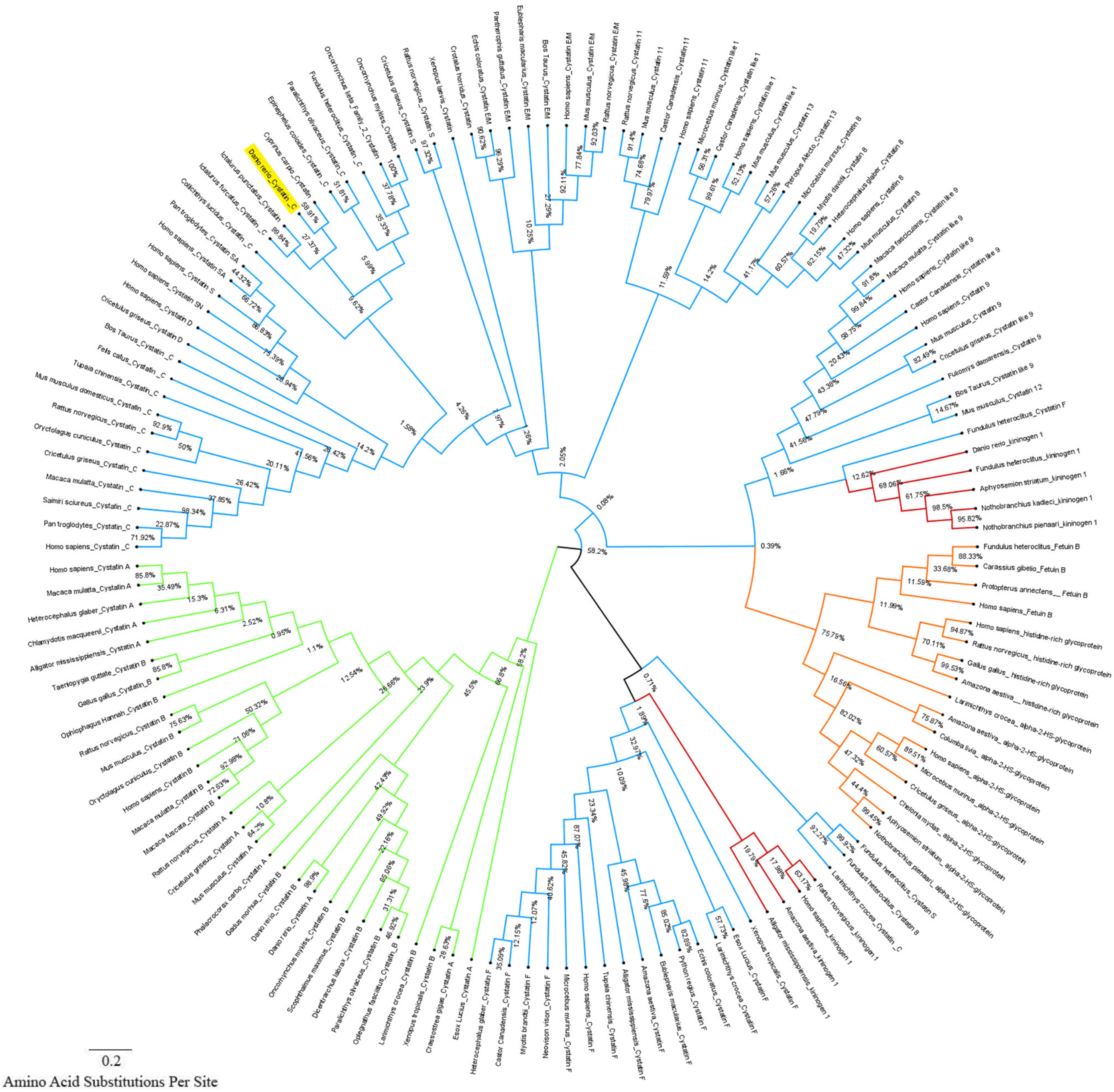

Supplementary Figure S1: Complete initial phylogenetic tree. Type 1 Cystatins in Green, Type 2 Cystatins in Blue, Type 3 Cystatins in Red and Type 4 Cystatins in Orange. The *Danio rerio* amino sequence highlighted yellow. This was filtered to formulate the final refined tree available in the main text.

```
MFLKIIVAFLAVILTVSSAGLVGGPTDADMDKDSESALQFAMAQYNRQSNDAYVRGVSKVTKLQKQVVAGIKYIFTVDVARTTCRKGGVEELCAIHENPEIAQVKECKIVVWTKLWENFIKVTENSCL
--HHHHHHHHHHHH----------EE-----HHHHHHHHHHHHHHHHHH-----EEEEEEEEEEEEEE----EEEEEEEEEEE---------------------EEEEEEEEEEEE----EEEEEE---
```

Supplementary Figure S2: JPred secondary structure prediction server result. The 'H' represents a helix, 'E' represents a beta sheet.

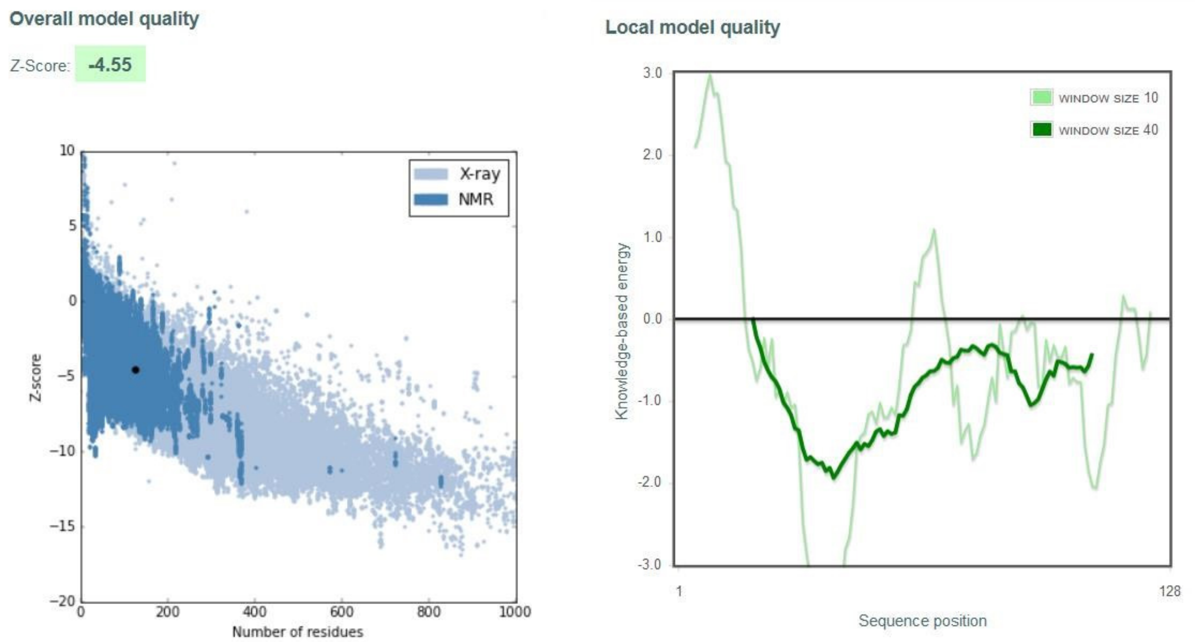

Supplementary Figure S3: ProSA-web service quality assessment plots for the I-Tasser generated model before refinement.

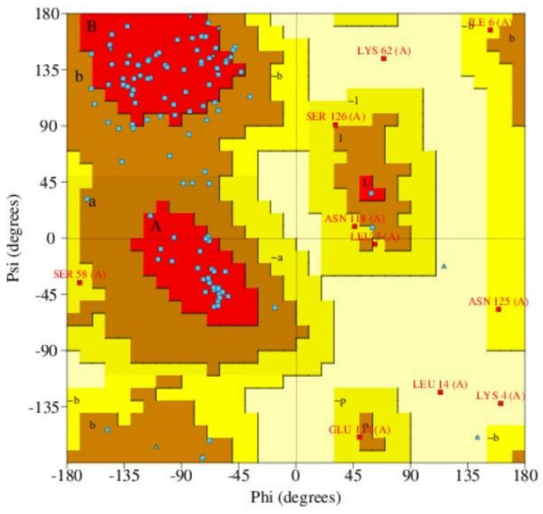

Ramachandran Plot statistics

		No. of residues	%-tage
Most favoured regions	[A,B,L]	79	67.5%**
Additional allowed regions	[a,b,l,p]	28	23.9%
Generously allowed regions	[~a,~b,~l,~p]	7	6.0%
Disallowed regions	[XX]	3	2.6%*
Non-glycine and non-proline residues		117	100.0%
End-residues (excl. Gly and Pro)		2	
Glycine residues		7	
Proline residues		2	
Total number of residues		128	

Supplementary Figure S4: Validation of the I-TASSER predicted protein structure using the Ramachandran plot before refinement.

Supplementary Table S1: Detailed description of the electrostatic charge and hydrophobic residue distribution on the protein surface of Cystatin C of *D. rerio*.

Patch	Type	Size (Å²)	Score	Intensity (Score / Size)
1	Positive	1024.2	866.788	0.846
2	Positive	43.4	38.615	0.89
3	Positive	15.4	0	0
4	Positive	672.4	597.782	0.889
5	Positive	21.5	17.476	0.811
6	Positive	18.4	7.222	0.393
7	Negative	120.9	115.675	0.957
8	Negative	60.1	77.194	1.284
9	Negative	86.8	105.921	1.221
10	Negative	251.3	281.105	1.119
11	Negative	79.9	80.66	1.01
12	Negative	104.4	154.691	1.482
13	Negative	23.4	24.225	1.035
14	Negative	206.6	253.733	1.228
15	Negative	41.3	34.583	0.837
16	Negative	29.9	21.893	0.732
17	Negative	25.6	12.23	0.477
18	Negative	17.9	13.604	0.76
19	Negative	176.2	251.095	1.425
20	Negative	25	32.309	1.294
21	Hydrophobic	1223.1	858.725	0.702
22	Hydrophobic	104.5	75.331	0.721
23	Hydrophobic	51.1	35.154	0.688
24	Hydrophobic	84.9	43.78	0.516
25	Hydrophobic	143	92.19	0.645
26	Hydrophobic	112.5	62.656	0.557
27	Hydrophobic	107.4	53.777	0.501
28	Hydrophobic	53.9	43.302	0.803

Supplementary Table S2: Protein Surface Analysis of the interacting surfaces between Cystatin C (A) and Papain (B). Details regarding the types of bonding including Hydrogen Bonding (HB), Salt Bridges (SB), Pi Stacking (Pi), Disulfide bonding (DS) and Vander Waal interactions (VW).

Residue	Closest	Distance (Å)	HB	SB	Pi	DS	VW
A:53:Tyr			0	0	0	0	0
A:58:Ser			0	0	0	0	0
A:59:Lys			0	0	0	0	1
A:78:Asp	B:156:Lys	2.7	0	1	0	0	11
A:80:Ala			0	0	0	0	0
A:83:Thr	B:64:Asn	2.1	0	0	0	0	9
A:84:Cyx			0	0	0	0	0
A:85:Arg			0	0	0	0	0
A:90:Glu			0	0	0	0	0
A:92:Leu	B:61:Tyr	2.2	0	0	0	0	1
A:93:Cyx			0	0	0	0	0
A:94:Ala			0	0	0	0	0
A:95:Ile	B:158:Asp	2.4	0	0	0	0	15
A:96:His	B:158:Asp	2.9	0	0	0	0	0
A:97:Glu	B:25:Cys<br>B:159:His<br>B:158:Asp<br>B:66:Gly	2.3<br>2.3<br>2.7<br>3.0	1	0	0	0	9
A:98:Asn	B:19:Gln	2.2	0	0	0	0	2
A:99:Pro	B:177:Trp<br>B:142:Gln	1.8<br>2.5	0	0	0	0	20
A:100:Glu			0	0	0	0	0
A:101:Ile			0	0	0	0	0
A:102:Ala			0	0	0	0	0
A:103:Gln			0	0	0	0	0

Supplementary Table S3: Protein Surface Analysis of interacting surfaces between Cystatin C (A), Cathepsin B (B). Details regarding the types of bonding including Hydrogen Bonding (HB), Salt Bridges (SB), Pi Stacking (Pi), Disulfide bonding (DS) and Vander Waal interactions (VW).

Residue	Closest	Distance (Å)	HB	SB	Pi	DS	VW
A:2:Phe			0	0	0	0	0
A:3:Leu	B:65:Ser	3.0 A	0	0	0	0	1
A:4:Lys			0	0	0	0	0
A:9:Phe			0	0	0	0	0
A:12:Val			0	0	0	0	0
A:43:Ala			0	0	0	0	0
A:44:Gln	B:196:Met	2.4 A	1	0	0	0	0
A:45:Tyr			0	0	0	0	0
A:47:Arg	B:196:Met	2.2 A	0	0	0	0	5
A:48:Gln			0	0	0	0	0
A:49:Ser			0	0	0	0	0
A:50:Asn			0	0	0	0	0
A:86:Lys			0	0	0	0	0
A:87:Gly			0	0	0	0	0
A:105:Lys			0	0	0	0	0
A:108:Lys			0	0	0	0	0

Supplementary Table S4: Protein Surface Analysis of interacting surfaces between Cystatin C (A), Cathepsin H (B). Details regarding the types of bonding including Hydrogen Bonding (HB), Salt Bridges (SB), Pi Stacking (Pi), Disulfide bonding (DS) and Vander Waal interactions (VW).

Residue	Closest	Distance (Å)	HB	SB	Pi	DS	VW
A:1:Met	B:21:Ser B:63:Cyx	1.3 A 2.6 A	0	0	0	0	21
A:2:Phe			0	0	0	0	0
A:3:Leu			0	0	0	0	4
A:4:Lys	B:142:Leu	1.9 A	0	0	0	0	21
A:7:Val			0	0	0	0	0
A:8:Ala	B:63:Cyx B:23:Gly	2.0 A 2.8 A	0	0	0	0	9
A:9:Phe	B:23:Gly B:158:Asn	2.7 A 2.9 A	0	0	0	0	13
A:10:Leu			0	0	0	0	0
A:12:Val			0	0	0	0	1
A:13:Ile			0	0	0	0	0
A:25:Pro			0	0	0	0	0
A:66:Gln			0	0	0	0	0
A:72:Lys			0	0	0	0	0
A:74:Ile			0	0	0	0	0
A:108:Lys			0	0	0	0	0
A:110:Val			0	0	0	0	0
A:124:Glu			0	0	0	0	0
A:125:Asn			0	0	0	0	0
A:126:Ser			0	0	0	0	0
A:127:Cyx			0	0	0	0	0
A:128:Leu			0	0	0	0	0

Supplementary Table S5: Protein Surface Analysis of interacting surfaces between Cystatin C (A), Cathepsin L1 (B). Details regarding the types of bonding including Hydrogen Bonding (HB), Salt Bridges (SB), Pi Stacking (Pi), Disulfide bonding (DS) and Vander Waal interactions (VW).

Residue	Closest	Distance (Å)	HB	SB	Pi	DS	VW
A:1:Met			0	0	0	0	0
A:2:Phe	B:66:Asn	2.1 A	0	0	0	0	5
A:3:Leu	B:66:Asn<br>B:65:Cyx<br>B:67:Gly	2.0 A<br>2.8 A<br>2.9 A	0	0	0	0	22
A:4:Lys	B:19:Gln	2.7 A	0	0	0	0	6
A:5:Ile			0	0	0	0	0
A:9:Phe	B:162:Asp	2.5 A	0	0	0	0	17
A:10:Leu			0	0	0	0	0
A:11:Ala			0	0	0	0	0
A:12:Val			0	0	0	0	0
A:13:Ile	B:21:Gln	2.0 A	0	0	0	0	6
A:14:Leu			0	0	0	0	0
A:16:Val			0	0	0	0	0
A:17:Ser			0	0	0	0	0
A:44:Gln			0	0	0	0	0
A:45:Tyr			0	0	0	0	0
A:48:Gln			0	0	0	0	0
A:72:Lys			0	0	0	0	0
A:110:Val			0	0	0	0	0
A:121:Lys			0	0	0	0	0
A:122:Val			0	0	0	0	0
A:123:Thr			0	0	0	0	0
A:124:Glu			0	0	0	0	2
A:125:Asn			0	0	0	0	0
A:126:Ser			0	0	0	0	0

Supplementary Table S6: Protein Surface Analysis of interacting surfaces between Cystatin C (A), Cathepsin S (B). Details regarding the types of bonding including Hydrogen Bonding (HB), Salt Bridges (SB), Pi Stacking (Pi), Disulfide bonding (DS) and Vander Waal interactions (VW).

Residue	Closest	Distance (Å)	HB	SB	Pi	DS	VW
A:1:Met	B:57C:Lys	2.5 A	0	0	0	0	5
A:2:Phe			0	0	0	0	0
A:3:Leu			0	0	0	0	0
A:8:Ala			0	0	0	0	0
A:9:Phe	B:113:Tyr	2.3 A	0	0	0	0	4
A:25:Pro			0	0	0	0	1
A:45:Tyr			0	0	0	0	0
A:48:Gln			0	0	0	0	0
A:61:Thr			0	0	0	0	0
A:62:Lys			0	0	0	0	0
A:64:Gln			0	0	0	0	0
A:66:Gln			0	0	0	0	0
A:72:Lys			0	0	0	0	0
A:74:Ile	B:61:Lys	2.2 A	0	0	0	0	12
A:75:Phe			0	0	0	0	0
A:76:Thr			0	0	0	0	1
A:105:Lys			0	0	0	0	0
A:106:Glu			0	0	0	0	0
A:108:Lys	B:61:Lys	2.8 A	0	0	0	0	13
A:124:Glu	B:67:Phe	2.4 A	0	0	0	0	3
A:125:Asn			0	0	0	0	0
A:126:Ser			0	0	0	0	0
A:127:Cyx			0	0	0	0	1
A:128:Leu			0	0	0	0	2

2. Analysis of Human and Rat AChE

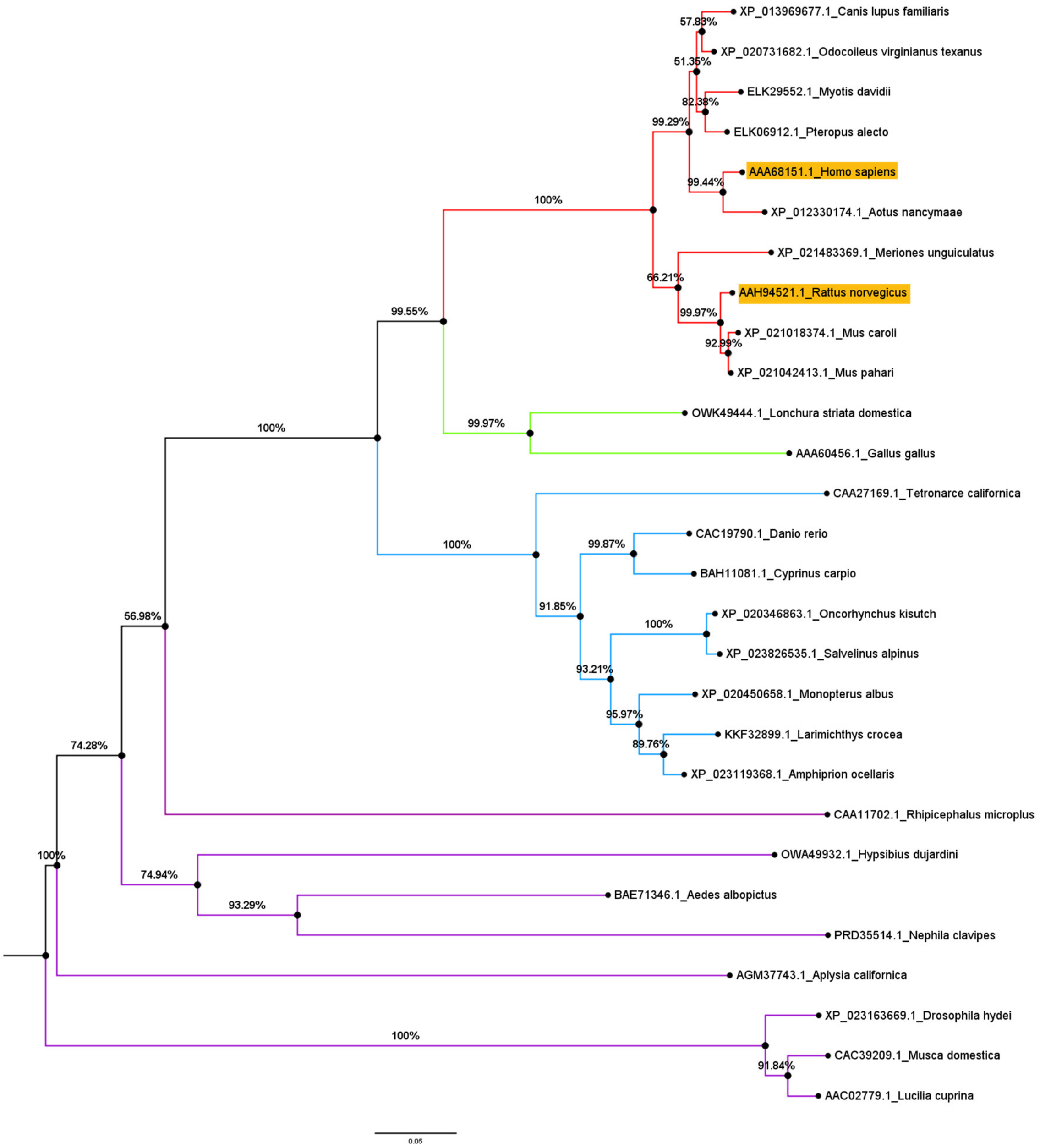

Supplementary Figure S5: Phylogenetic Tree Analysis of Acetylcholinesterase. Clear distinct clade separation represented as follows with Mammalian Ache in Red, Aves Ache in Green, Pisces in Blue and Invertebrate Ache in Purple. *Homo sapiens* and *Rattus norvegicus* highlighted in yellow.

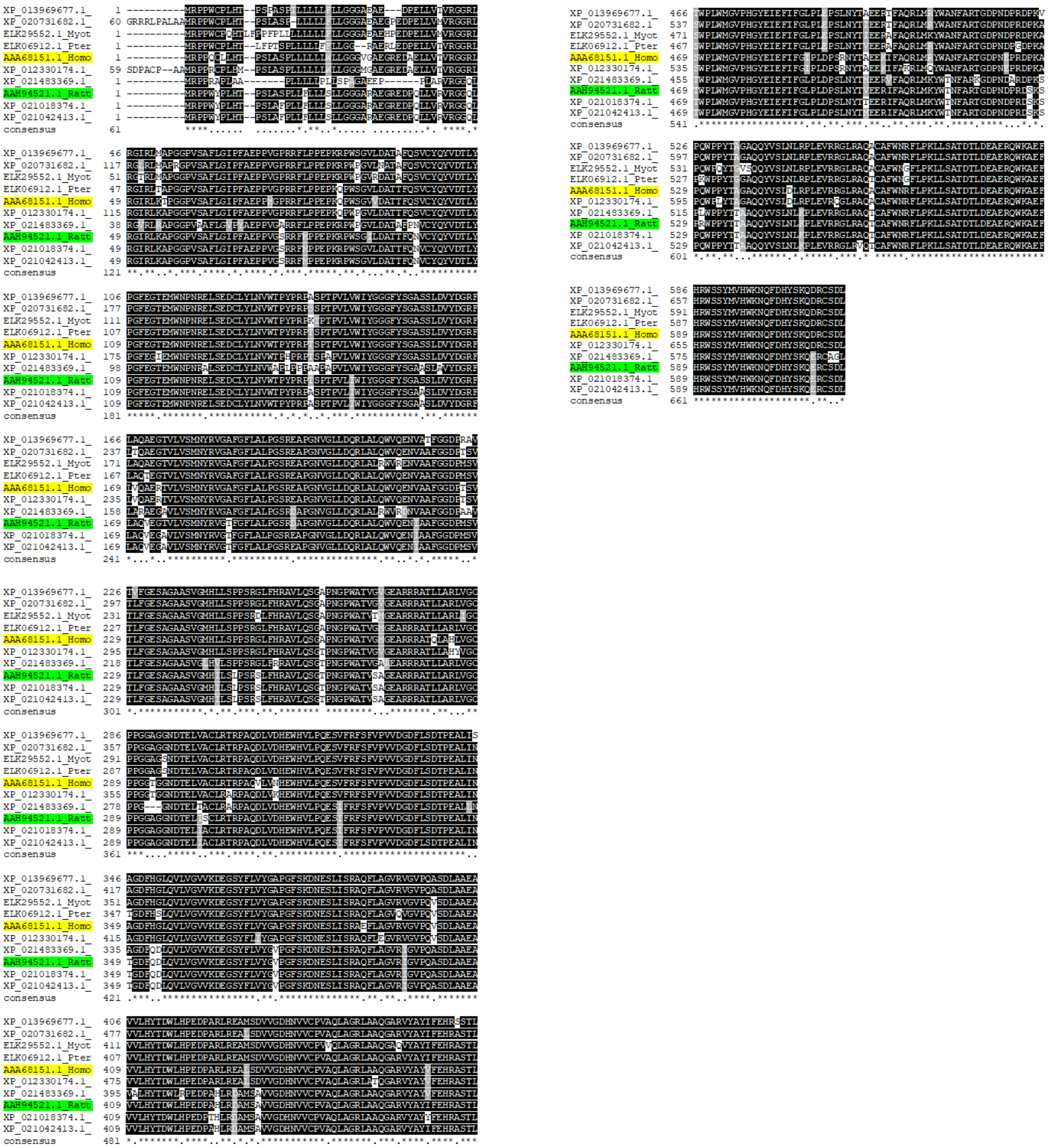

Supplementary Figure S6: Multiple Sequence Alignment by ClustalW of mammalian Ache. Symbol * depicts highly conserved sequence motifs. *Homo sapiens* sequence highlighted in yellow and *Rattus norvegicus* highlighted in green.

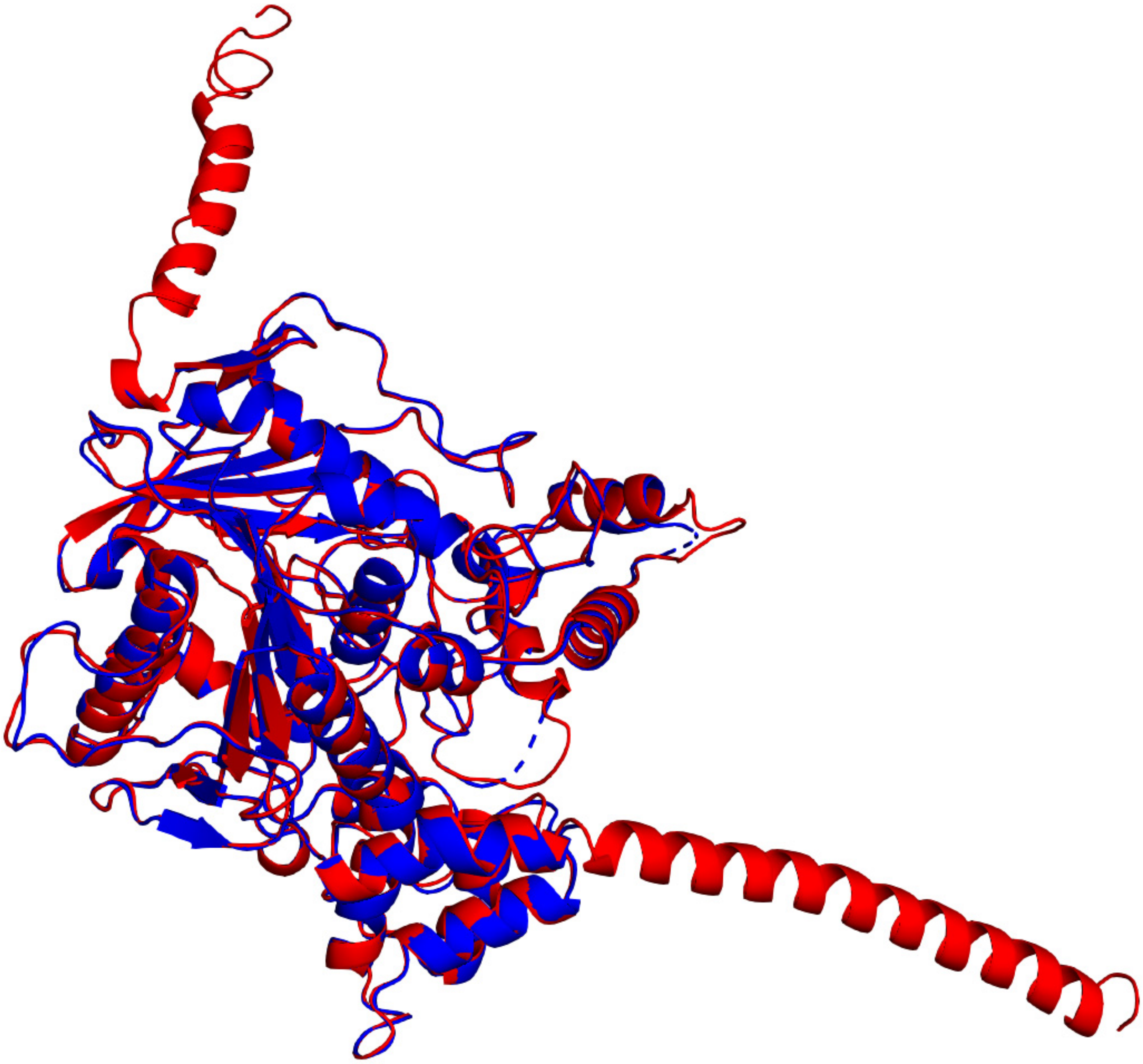

Supplementary Figure S7: The three-dimensional alignment of the AChE crystal structure of *Rattus norvegicus* (red) against the AChE crystal structure of *Homo sapiens* (blue).

Supplementary Table S7: AutoDock Vina results for protein ligand docking between hAChE and Echothiophate.

Mode	Affinity (kcal/mol)	Distance from best mode rmsd l.b.	rmsd u.b.
1	-5.6	0	0
2	-5.5	1.91	3.012
3	-5.5	3.554	5.917
4	-5.4	1.434	3.414
5	-5.3	3.056	5.733
6	-5.2	3.6	5.82
7	-4.9	2.177	3.504
8	-4.8	3.555	5.875
9	-4.7	2.272	3.646

Supplementary Table S8: AutoDock Vina results for protein ligand docking between rAChE and Echothiophate.

Mode	Affinity (kcal/mol)	Distance from best mode	
		rmsd l.b.	rmsd u.b.
1	-4.7	0	0
2	-4.5	1.906	2.462
3	-4.5	3.9	4.962
4	-4.5	3.912	4.817
5	-4.4	1.957	2.752
6	-4.4	1.951	2.9
7	-4.4	1.099	3.189
8	-4.3	3.788	4.826
9	-4.1	1.58	3.36

Supplementary Table S9: AutoDock Vina results for protein ligand docking between hAChE and the negative control Imidazole.

Mode	Affinity (kcal/mol)	Distance from best mode	
		rmsd l.b.	rmsd u.b.
1	-3.3	0	0
2	-3.3	16.04	16.42
3	-3.2	16.927	17.148
4	-3.2	11.862	12.551
5	-3	16.012	16.246
6	-3	11.788	12.15
7	-3	16.386	16.685
8	-3	17.025	17.716
9	-2.9	16.189	16.412

Supplementary Table S10: AutoDock Vina results for protein ligand docking between rAChE and the negative control Imidazole.

Mode	Affinity (kcal/mol)	Distance from best mode	
		rmsd l.b.	rmsd u.b.
1	-3.2	0	0
2	-3.2	12.014	12.21
3	-3.1	13.551	13.739
4	-3.1	16.373	16.682
5	-3.1	15.291	15.815
6	-3	17.291	17.651
7	-3	12.631	13.237
8	-3	5.996	6.58
9	-2.8	16.366	16.565

3. Docking analysis between Survivin protein and 4-hydroxypyridine 1-oxide pyridin-4-ol 1-oxide

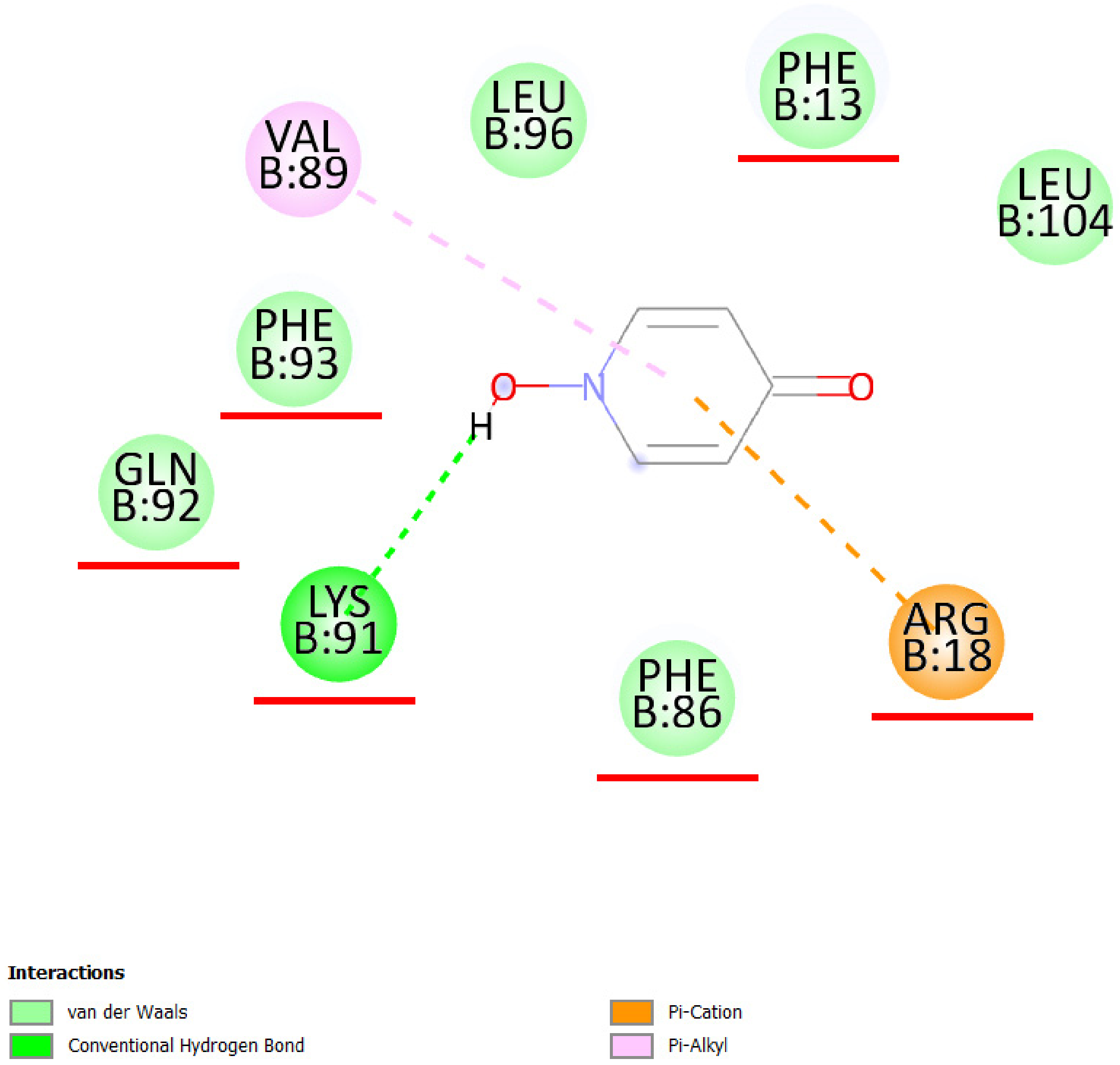

Supplementary Figure S8: The 2D representation of the protein-ligand complex formed in the Survivin protein and 4-hydroxypyridine 1-oxide pyridin-4-ol 1-oxide. The amin acid residues that were present in the original study have been marked by a red underline.

Supplementary Table S11: AutoDock Vina results for protein ligand docking between Survivin protein and 4-hydroxypyridine 1-oxide pyridin-4-ol 1-oxide.

Mode	Affinity (kcal/mol)	Distance from best mode	
		rmsd l.b.	rmsd u.b.
1	-4.6	0	0
2	-4.5	1.799	2.119
3	-4.5	3.621	4.458
4	-4.4	1.973	3.053
5	-4.3	1.095	3.186
6	-4.3	3.834	4.07
7	-4.2	3.581	4.581
8	-4.1	3.174	4.023
9	-4.1	3.626	4.078

4. Docking analysis between Haemoglobin and Heam.

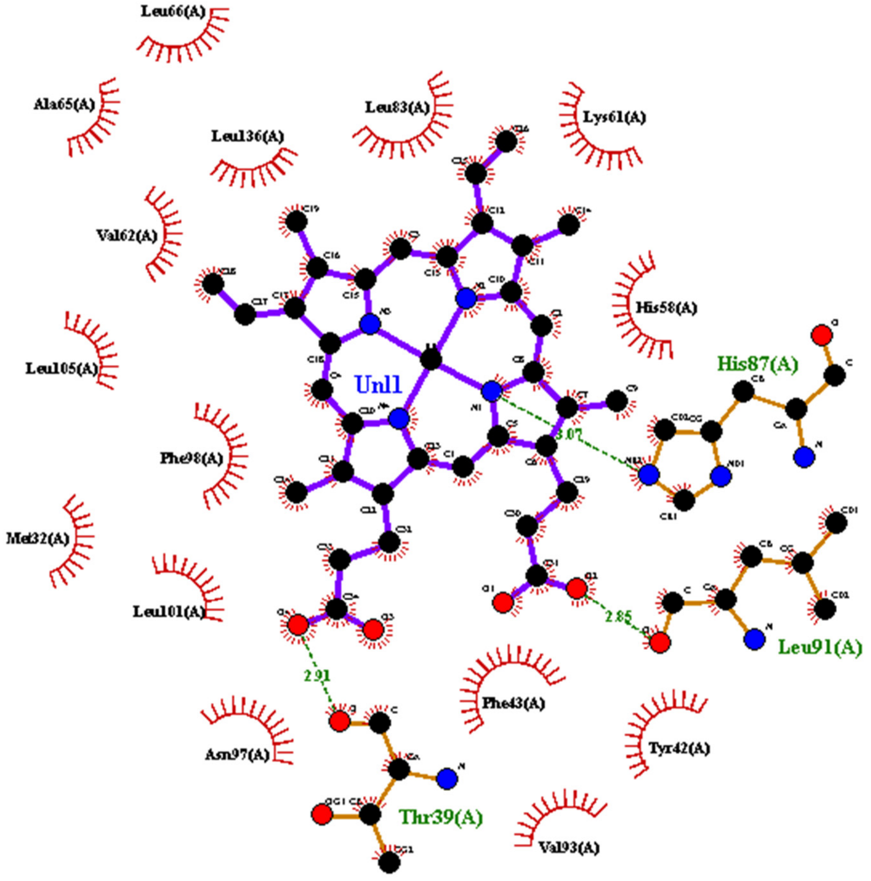

Supplementary Figure S9: The 2D representation of the protein-ligand complex formed in the Hemoglobin and the heam ligand. The characteristic "heme coordinated to the histidine residue" protein-ligand interaction can be seen between the heam ligand's central iron atom and the Haemoglobin's Histidine amino acid.

Supplementary Table S12: AutoDock Vina results for protein ligand docking between Haemoglobin and Heam.

Mode	Affinity (kcal/mol)	Distance from best mode	
		rmsd l.b.	rmsd u.b.
1	-11.4	0	0
2	-11.3	2.448	6.798
3	-11.1	2.469	6.164
4	-11.1	0.357	6.347
5	-10.8	4.346	9.924
6	-9.7	2.932	6.106
7	-9.7	4.683	10.216
8	-8.8	4.031	8.964
1	-11.4	0	0
